# Predictions of DNA mechanical properties at a genomic scale reveal potentially new functional roles of DNA flexibility

**DOI:** 10.1093/nargab/lqad097

**Published:** 2023-11-06

**Authors:** Georg Back, Dirk Walther

**Affiliations:** Max Planck Institute of Molecular Plant Physiology, Am Mühlenberg 1, Potsdam-Golm 14476, Germany; Max Planck Institute of Molecular Plant Physiology, Am Mühlenberg 1, Potsdam-Golm 14476, Germany

## Abstract

Mechanical properties of DNA have been implied to influence many of its biological functions. Recently, a new high-throughput method, called loop-seq, which allows measuring the intrinsic bendability of DNA fragments, has been developed. Using loop-seq data, we created a deep learning model to explore the biological significance of local DNA flexibility in a range of different species from different kingdoms. Consistently, we observed a characteristic and largely dinucleotide-composition-driven change of local flexibility near transcription start sites. In the presence of a TATA-box, a pronounced peak of high flexibility can be observed. Furthermore, depending on the transcription factor investigated, flanking-sequence-dependent DNA flexibility was identified as a potential factor influencing DNA binding. Compared to randomized genomic sequences, depending on species and taxa, actual genomic sequences were observed both with increased and lowered flexibility. Furthermore, in *Arabidopsis thaliana*, mutation rates, both *de novo* and fixed, were found to be associated with relatively rigid sequence regions. Our study presents a range of significant correlations between characteristic DNA mechanical properties and genomic features, the significance of which with regard to detailed molecular relevance awaits further theoretical and experimental exploration.

## Introduction

The structure and mechanical properties of double-stranded DNA (dsDNA) have been of interest since its discovery in 1869 (1). Since then, besides its primary double-stranded helical structure, also known as B-DNA, a variety of different secondary structures of DNA have been discovered. Different helical forms, such as A-DNA and Z-DNA, DNA G-quadruplex and cruciform-DNA have been shown to have a wide range of biological functions (2–5). In addition to the existence of distinct conformational variants, basic mechanical properties of the dominating B-form of genomic DNA influence many aspects of genomic DNA maintenance within cells, its functions, and interactions with other molecules, mainly proteins. The eukaryotic genome is packaged in the form of nucleosomes, which is formed by a ∼150 bp stretch of DNA wrapped around a histone protein complex. Nucleosome formation has been linked to flexibility of genomic DNA, with histones binding to more flexible DNA regions, while linkers and nucleosome-depleted regions (NDR) have been reported to be more rigid (6–8). Specifically, NDRs have been connected to long poly-dA:dT tracts with rigid structure resistant to sharp bending (9–11). Variable DNA flexibility has also been discussed in the context of nanotechnological applications (12).

With regard to interactions of genomic DNA with proteins, transcription factor (TF) binding has long been considered connected to DNA shape. Both, detection capabilities of proteins for specific shape properties of DNA as well as bending of the DNA upon protein binding have been reported (13–15), reviewed in (16). A recent publication succeeded in predicting DNA-protein interaction by explicitly considering DNA shape as a determinant feature (17). A puzzling observation with regard to TF-binding to genomic regions has been that some loci with canonical TF-binding motifs present were found to be bound by TFs, while others were not. Recently, it has been suggested that the flanking regions of the binding motif of TFs are key in determining binding efficiency. Since there is more sequence variation in these flanking regions than in the consensus motifs, it has been suggested that mechanical properties, specifically their flexibility, may play a major role in defining the binding site (18–20).

The mechanical flexibility of dsDNA is determined by the constraints of its backbone, high charge density (negatively charged phosphate groups), as well as its sequence of bases and their stacking interactions (21,22). dsDNA was considered among the most rigid biopolymers with a persistence length of ∼50 nm, or approximately 150 bp (21). However, recent studies showed that short DNA sequences under 100 bp are able to loop spontaneously, indicating that DNA is much more flexible on a short scale than previously assumed (23–25). The underlying sequence determinants have hence been intensively researched, for review see (26).

Based on the potential for looping of short DNA sequences, several assays for measuring DNA flexibility of short sequences have been developed. For a recent review, see (27). Loop-seq, developed recently by Basu *et al.* (28), uses a sequencing-based approach, allowing a high-throughput measurement of DNA mechanical properties. For this method, a library of DNA fragments flanked by two adapter sequences is immobilized and nicked. Then, the sequences are allowed to loop for a specific amount of time. Afterwards, unlooped DNA is digested, the library is amplified, sequenced and compared to a control treated identically, but without digestion. Overrepresented sequences have a short looping time, meaning they are more flexible, while underrepresented sequences are more rigid. The natural logarithm of the ratio of the relative population of a sequence in the sample pool to that in the control was termed ‘cyclizability’, with a low value representing rigid, and a high value flexible sequences.

The study by Basu *et al.* (28) confirmed many previously observed effects of DNA mechanics, such as being a key factor in nucleosome positioning. In addition, they were able to show that codon usage might be influenced by DNA mechanics. Furthermore, their study also provided a large dataset linking sequence and measured cyclizability, allowing the development of machine learning prediction tools. An accurate computational prediction model may find many applications and may even potentially replace experimental methods, while also leading to a higher base-pair resolution. Accordingly, several attempts have been made and high prediction accuracies have been reported (29,30).

In a follow-up study, Basu and co-workers (31) further investigated the sequence-flexibility relationships by developing a prediction model that explicitly builds in surmised relevant features, such as dinucleotide frequencies and their respective pairwise distances, revealing the relevance of dinucleotides as a determining factor of local DNA flexibility. Also, by including additional experimental data, they showed that DNA methylation generally leads to a stiffening of DNA.

In this study, a deep learning CNN/LSTM model, CycPred, was developed and utilized to further explore the biological functions of local DNA flexibility. Our work builds on the previously published studies on experimentally measured (28) and computationally predicted DNA cyclizability scores (29–31). We investigated the potential significance of mechanical flexibility in several species from different kingdoms, including plants, with regard to magnitude relative to random expectation, underlying sequence determinants, sequence range, and genomic features, such as transcription start sites, TF-binding sites, single nucleotide polymorphisms (SNPs) and *de novo* mutations, DNA-methylation-, and crossing-over sites. We show that mechanical properties may indeed play a crucial role in determining the sequence-structure-function relationships of genomic DNA.

## Materials and methods

### Utilized datasets

For training and validating the computational prediction model, the datasets for intrinsic cyclizability in yeast as reported by Basu *et al.* (28) were utilized. All intrinsic cyclizability datasets consisted of sequences of length 100 bp, of which 50 bp are actual probe-sequence, with 25 bp flanking sequences on either side with their respective cyclizability score, based on loop-seq experiments. The utilized sequences were the 82 404 probe-sequence windows with step size 7 bp for chromosome V, 19 907 sequences for the nucleosome library, 82 368 sequence windows with step size 7 bp for the nucleosome tiling library, and 12 472 sequences for the library containing random sequences. The dataset of 92 807 sequences with the respective cyclizability score in unmethylated and CpG methylated context were reported in a recent study by Basu *et al.* (31).

The *Saccharomyces cerevisiae* genome S288C and its annotation was downloaded from the SGD database https://www.yeastgenome.org/ (32). The genome of *Arabidopsis thaliana*, TAIR10, and its annotation was obtained from https://www.arabidopsis.org/ (33). The genome and annotation of *Chlamydomonas reinhardtii* was downloaded from Phytozome (https://phytozome-next.jgi.doe.gov/) (34). The genomes and annotations of all other species were obtained from https://www.ncbi.nlm.nih.gov/.

*Arabidopsis thaliana* nucleosome position and occupancy data was based on MNase-seq data by Jiming Jiang *et al.* (35), downloaded from PlantDHS (http://plantdhs.org/) (36).

Methylation calls for *Arabidopsis thaliana* were taken from the 1001 genome project (37), as were the SNP and short indel calls (38). With regard to *de novo* mutations, calls as reported by Monroe et al. (39) were utilized.

Transcription factor binding site (TFBS) motifs were obtained from JASPAR (40), and DAP-seq data, indicating transcription factor binding events to ‘naked’ DNA, for *Arabidopsis thaliana* from O’Malley *et al.* (41).

The Spo11-oligo-seq Arabidopsis dataset utilized in this study was published by Choi et al. (42) and kindly provided by the study authors. It is reported in the from of log2(spo11-oligonucleotides/gDNA), with spo11-olignucleotides representing number of reads mapped at a genomic position, and gDNA representing number of single-end reads from genomic DNA mapped to the same position as control. The data was mapped to the Col-Cen genome (43), which was obtained from https://github.com/schatzlab/Col-CEN.

### Design and training of the deep learning model

**Figure 1.**
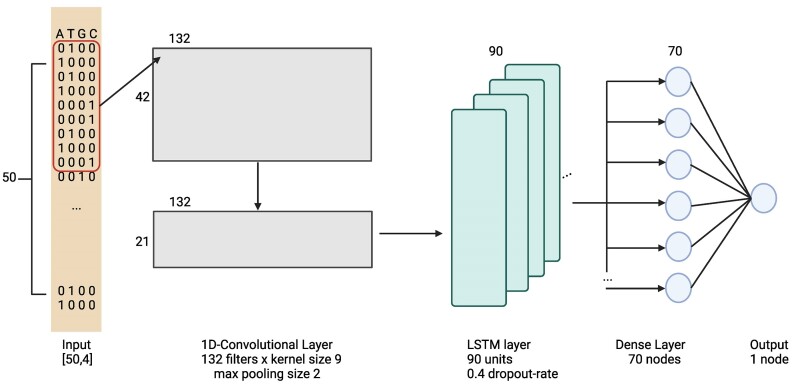
Architecture of the prediction model. Input sequences of length 50bp are one-hot encoded and passed through a neural network with sequential layers of a 1D-convolutional and LSTM layers ReLU activation function. Finally, a fully connected layer with ReLU activiation leads to the output node with linear activation. ‘Adam’ was used as the minimizer. Kernel sizes, number of filters, and number of units and nodes are given as labels in the schema. The schematic representation of the model was created with BioRender.com.

The Python package tensorflow-gpu 2.6 was used to create the deep learning model for predicting the measured cyclizability values (28) based on DNA-sequence (Figure 1). For optimizing the model architecture, the package kerastuner 1.04 was utilized.

In all intrinsic cyclizability datasets, adapter sequences (25 bases at either terminus) were removed, resulting in sequences of length 50 bp. The tiling library was split into training and test sets with a 70% / 30% split. As the dependent variable, the intrinsic cyclizability score denoted as C0 was used as the target variable. As the loss function, the mean squared error (MSE) was applied. The model utilizes 1D convolution layers with max pooling, followed by an LSTM layer with dropouts. The last hidden layer is a dense layer followed by the output layer (Figure 1). Due to the discrepancy between cyclizability of forward and reverse sequences, which was also reported in the original paper (28), both the forward and reverse-complement of each sequence windows were predicted and the mean was reported as cyclizability value.

For identification of potential motifs identified by the model, we used DeepLIFT (44) for calculation of importance scores, and TF-MoDISco (45) for the identification of potential motifs based on those importance scores.

### Calculation of Cohen's d effect size

Cohen's d effect sizes were calculated as follows:


(1) \begin{equation*}d = \frac{{\ {\bar{x}}_1\ - \ {\bar{x}}_2}}{s}\ \hbox{with }s = \sqrt {\frac{{\left( {{n_1} - 1} \right)s_1^2 + \left( {{n_2} - 1} \right)s_2^2}}{{{n_1} + {n_2} - 2}}} ,\end{equation*}

where ${\bar{x}}_{1/2}$ are the respective means of two distributions 1 and 2, s_1/2_ are the associated standard deviations, and n_1/2_ their respective numbers of observations.

### Analysis of sequences around transcriptions start sites (TSSs)

TSS positions were determined using available genome annotations (gff files), with the position of the first base of the 5′ UTR taken as the TSS. For each gene, only one isoform, isoform 1, if available, was chosen.

Starting from position -525 bp (with the negative sign indicating position towards the 5′ of DNA, i.e. ‘upstream’) from the TSS and ending at position +525 bp, the 1050 bp long sequence results in 1000 windows of length 50 bp. For each window, cyclizability was predicted using the trained model. Each position in the respective profile plots represents the mean of all windows with this position as their 25th base, where the mean is taken across all determined TSSs in the respective genome.

For grouping genes into TATA-box-containing genes and genes without TATA-box, we used definitions as described previously (46).

For investigating the effects of nucleotide/dinucleotide composition on the cyclizability values, we applied a shuffling and a sequence sampling approach. For shuffling, each above-mentioned 50 bp sequence window was either randomly shuffled at the single-nucleotide level or shuffled with the fasta-dinucleotide-shuffle algorithm (47). In essence, sequences were shuffled ‘horizontally’. Alternatively, for sampling, the nucleotide- and dinucleotide-probability at each position of the 1050 bp long sequences were calculated based on actual sequences around all TSSs. Nucleotide-based sampling involves simply drawing nucleotides according to their probability at each position. For dinucleotide sampling, a random sequence was generated by adding nucleotides to a growing sequence, initialized by a single base. Added nucleotides are drawn based on the conditional probability of the four nucleotides on the preceding base, initialized at the first position according to the observed base probability at this site. Conditional probabilities were determined based on all actual sequences around TSSs. The latter can be referred to ‘vertical sampling’ across all TSS-centered sequences with consideration of conditional probabilities, similar to a first-order hidden Markov model.

Dinucleotide shuffling puts higher constraints on the sequence than random shuffling at the level of single bases, resulting in dinucleotide-shuffled sequences having, on average, a higher sequence identity to the original sequence. To account for that we employed a modified shuffling method (horizontal shuffling), in which, given a set of sequences to shuffle, we first created a dinucleotide-shuffled version of each sequence in the set and calculated the sequence distance as judged by the number of matching positions between those sequence pairs. We then shuffled each original sequence at the level of single bases by repeatedly exchanging two randomly selected bases in the sequence until the resulting sequence had the same sequence distance to the original sequence as the dinucleotide-shuffled version. Thus, both the dinucleotide-shuffled and mono-nucleotide-shuffled sequences were equally distant (sequence-wise) from a given initial sequence, allowing a comparison of their respective cyclizability and accounting for differences in sequence constraints. For both randomized sequence versions, their respective cyclizability values were predicted and the difference to the common parent-sequence was recorded.

### Statistical analysis of trends in cyclizability at a genomic scale

For each organism, we tiled the respective genomes into sequence windows of length 50 bp at a 50 bp increment (step size). Windows containing unresolved bases (IUPAC ambiguity codes) were discarded. Each of the windows was randomly sequence-shuffled horizontally five times respectively, and the difference between the prediction of the actual genomic sequence and the predictions of the shuffled sequences was reported. A resulting value below zero indicates that the genomic sequence has a lower predicted cyclizability, and is thus ‘stiffer’ than expected for randomly shuffled versions of the same sequence, a value above zero indicates the opposite, i.e. more flexible than expected.

### Analysis of *de novo* mutations, fixed SNPs and methylated positions in *Arabidopsis thaliana*

For each DNA-methylation sequence context (CG, CHG, CHH), all sequence windows of length 50 bp containing a C-position corresponding the respective sequence context and observed to become methylated and located in promoter regions (defined as 300 bp sequence window upstream of a TSSs) were compared to all promoter windows containing unmethylated versions of the same context with regard to their predicted cyclizability.

For both naturally occurring (fixed) SNPs and *de novo* mutations, all windows with a *de novo*/ fixed SNP were sampled, their cyclizability was predicted and compared to the predicted cyclizability of all windows with no reported mutation in the respective dataset.

### Analysis of cyclizability in relation to transcription factor (TF) binding

TF binding was analyzed by comparing bound sequence motifs in the promoter region, defined as 1000 bp upstream of the TSS, to unbound occurrences of the same motif. A TF motif was considered ‘bound’ when there was an overlap between DAP-seq region reported for the respective TF (41) and a FIMO (48) search with the motif-PWM (position weight matrix) associated with the respective TF with a *q*-value < 0.1, and unbound otherwise.

### Analysis of Spo11 oligonucleotides

Spo-11 oligonucleotide mapping in Arabidopsis were taken from Choi *et al.* (42). For each non-zero value of log_2_(spo11-olignucleotide/gDNA), provided on a per-base basis, and excluding the centromere region on Arabidopsis chromosome 1, left arm, interval base positions 1 bp:13 Mb, cyclizability for a 50 bp window centered on the respective position was predicted. The Col-Cen reference genome as downloaded from (https://github.com/schatzlab/Col-CEN), to which the Spo11 data was mapped, was utilized. Pearson correlation coefficients between Spo11 values and predicted cyclizabilities were calculated.

## Results

We first report on the performance of our computational prediction method with regard to predicting cyclizability, and thus local genomic DNA mechanical flexibility/ rigidity. After having established high accuracy and examining the underlying sequence-determinants of cyclizability, we apply the model to investigate differences between the genomes of several species from different kingdoms and the potential relevance of local DNA rigidity on a range of different phenomena, such as transcription initiation and transcription factor binding, specific genomic sites including methylation sites, sequence variants, double-strand DNA breaks, and with a particular focus on the genome of the plant *Arabidopsis thaliana*, for which rich annotation and additional genomic-variant information is readily available. Of note, the actual target variable, on which the model was trained, is ‘cyclizability’, i.e. the ability to form closed-loops. As we take this parameter as a surrogate of ‘flexibility’, we use this term, as well as its logical complement, ‘rigidity’.

### The CNN/LSTM-based CycPred model

Our computational prediction model, termed CycPred, uses 1D Convolutional neural network (CNN) layers with max pooling followed by an LSTM layer and two fully-connected dense layers (Figure 1). The model DNAcycP by Li *et al.* (29), published during our work on this project and with identical objective and underlying input datasets, has a similar architecture, utilizing a CNN based Inception-ResNet structure and an LSTM layer.

Best performances were obtained when trained on the tiling-library (28), also when applied to other datasets (i.e. random, yeast chromosome V) and also better than models trained on those particular datasets. DNAcycP and our model performed equally well. All analyses reported in this study were performed using our prediction model CycPred. In the case of prediction of the 12 742 sequences in the random library, both yielded a correlation between predicted and actual cyclizability score of 0.93 (Pearson correlation coefficient), with a median distance between predicted and actual score of 0.092 for DNAcycP and 0.089 for our model. In case of the chromosome V library data, both models show a correlation of 0.77, with DNAcycP having a median distance of 0.176 and our model of 0.178.

Recently, Basu and co-workers published an new loop-seq experimental dataset using sequences derived from the mouse genome (31). They created two libraries, one containing 92 807 unmethylated sequences and another containing CpG methylated versions of the same sequences. Predicted values of our model showed a correlation (Pearson correlation coefficient, *r*) to the unmethylated dataset of *r* = 0.69, and 0.77 for the methylated dataset, with median distance between predicted and experimental cyclizability values of 0.212 and 0.159, respectively. These results are comparable to the prediction accuracy achieved for the yeast chromosome V library, showing that the model is able to generalize. Interestingly, our model seemed to be more accurate for the methylated library, which CycPred was not trained on. To investigate this further, we also trained models with the methylated and unmethylated mouse datasets, respectively, with the same architecture as used for the base-CycPred model. We observed that the models showed very little difference in prediction, with both models having a higher correlation between predicted and actual values for the methylated dataset (*r* = 0.75, *r* = 0.78) than for the unmethylated dataset (*r* = 0.67, *r* = 0.67).

### Pronounced changes of cyclizability around transcription start sites

The study by Basu *et al.* (28) showed that in yeast, sequence windows located approximately 150 bp upstream of the so-called ‘+1 dyad’, i.e. the position of the first location of high nucleosome occupancy downstream of the TSS, has a pronounced lower cyclizability relative to neighboring sequence locations. Given that this region of significantly lowered nucleosome occupancy is located approximately 20–50 bp upstream of TSSs, this result was interpreted as an indication of genomic DNA being rigid in locations of low nucleosome occupancy, so-called nucleosome depleted regions (NDRs), thereby facilitating access of transcription factors to their respective target sites in gene promoters, an observation that agreed with previous considerations to this effect (49). By also developing and applying a computational prediction tool, Li *et al.* (29) were able to demonstrate that also in mouse, nucleosome positions in general coincide with increased flexibility. However, unlike in yeast, where a rigid region was observed upstream of the TSS, in mouse, a rigid region was found downstream of the TSS.

**Figure 2.**
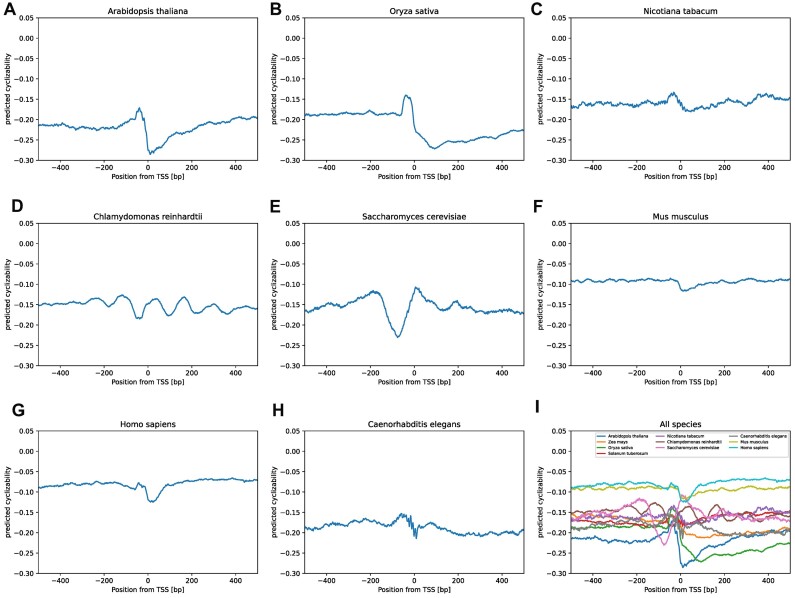
Comparison of predicted cyclizability around transcription start sites (TSSs) of different species. Mean predicted cyclizability around the TSS of all annotated genes containing a 5′ UTR of **(A)***Arabidopsis thaliana*, **(B)***Oryza sativa*, **(C)***Nicotiana tabacum*, **(D)***Chlamydomonas reinhardtii*, **(E)***Saccharomyces cerevisiae*, **(F)***Mus musculus*, **(G)***Homo sapiens*, **(H)***Caenorhabditis elegans*, **(I)** summary of all species.

To further investigate the potential role of DNA mechanical properties in transcription initiation, we examined the characteristic profile of cyclizability around TSSs in eight different species, three plants (*Arabidopsis thaliana* (Ath), *Orzya sativa* (Osa), *Nicotiana tabacum* (Nta)), one single-cell alga (*Chlamydomonas reinhardtii* (Cre)), yeast (*Saccharomyces cerevisia*e (Sce)), two mammals (*Mus musculus* (Mmu), *Homo sapiens* (Hsa)), and the invertebrate *Caenorhabditis elegans* (Cel) (Figure 2). Noticeably, in all species pronounced changes of cyclizability near the TSS are apparent. Six of the eight species (all but Sce and Cre) show very similar profiles with a pronounced and TSS-downstream-sided dip of cyclizability. In yeast (Sce), a pronounced dip was found ∼0–150 bp upstream of the TSS, thus approximately where it had been reported previously (28,50). In addition to the observed dips, regions of increased cyclizability, noticeably flanking the dips, are also apparent (upstream of the dip: Ath, Osa, Nta, Cel or symmetrical around it: Sce). The pattern of change of cyclizability in the alga Cre stands out, exhibiting a cyclical behavior.

### Autocorrelation of cyclizability shows no large-scale patterns

**Figure 3.**
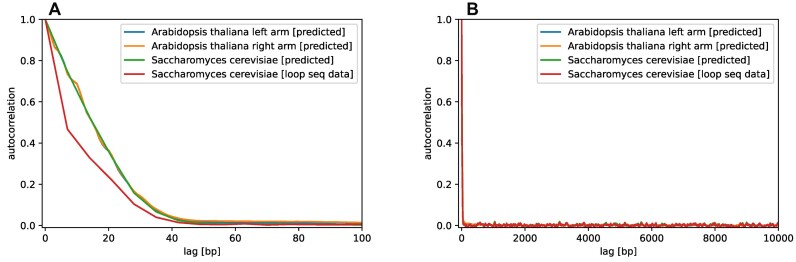
Autocorrelation shows no long-range patterns. Autocorrelation function (ACF) of predicted cyclizability *Arabidopsis thaliana* chromosome 1 (10 Mb segment on left arm (positions 1–11 Mb) and right arm (positions 16–26 Mb) to avoid the centromer with unavailable sequences), window size 50 bp, step-size 1 bp, **(A)** with maximum lag of 10 000 bp, **(B)** with a maximum lag of 100 bp. Also included are ACF values obtained on loop-seq-experimentally determined cyclizabilities of chromosome 5, *Saccharomyces cerevisiae* (cyclizability values at 7 bp resolution, (28)) as well as corresponding predicted values using CycPred (Pearson correlation coefficient, *r*, between predicted and observed values: *r* = 0.77). Notably, the ACF values of the experimental set decay even faster, possibly explained by experimental variance, smoothed out by the CycPred prediction model.

As we observed a characteristic and pronounced change of cyclizability locally around TSSs (Figure 2), we tested whether cyclizability exhibits long-distance patterns in the genome. Using the genome of *Arabidopsis thaliana* as a test species, we computed the autocorrelation function (ACF) of cyclizability for chromosome 1. With autocorrelation, the persistence length of similar cyclizability as well as the presence of periodic patterns would be detectable. However, there was no long-range pattern discernible (Figure 3A, B). ACF-values decreased rapidly with positional distance (lag) from the reference position, dropping off to near zero shortly before 50 bp, incidentally the size of windows used to predict local cyclizability. Performing an autocorrelation analysis on the previously published experimental cyclizability values of a 7 bp-resolution tiling sequence set of chromosome V of *Saccharomyces cerevisiae* (28), both experimental and predicted values, yielded the same results (Figure 3B). Notably, the ACF values of the experimental set decay even faster, possibly explained by experimental variance, smoothed out by the CycPred prediction model. This shows that due to high local variance, there is no longe-range persistence length of local cyclizability (see further discussion on this point below).

### Dinucleotide composition is a driving factor of averaged local DNA-rigidity profiles

Previous studies (29,30) as well as the model created in this study showed a high accuracy of computationally predicting cyclizability values of the loop-seq data (*r* = 0.94, Pearson correlation of actual versus observed cyclizability), suggesting that there is a set of ‘rules’ determining the rigidity of DNA that the model was able to learn and that rigidity is determined by sequence. Previous studies concluded that base composition is not a good indicator of DNA rigidity, except for mono-base or two-base repeats (51). This was confirmed by the two existing computational models (29,30) as well as by our model, CycPred. Shuffling random sequences (each base assumed with equal frequency (25%) and shuffling at the level of individual bases) leads to a large variance of predictions. Performing 1000 repeats of shuffling of random sequences, 1000 times, respectively, led to a median standard deviation of 0.313 of predicted cyclizability. However, when shuffling at the level of dinucleotides, performing the same experiment on the same set of random sequences, yielded a significantly different set of standard derivations (*P* < 0.0001, Cohen's *d* = –0.64), with a median of 0.273, and thus significantly smaller than obtained for single-base-level shuffled sequences. In part, this difference may be explained by a higher sequence similarity of the dinucleotide-shuffled sequence to an original sequence vs. a randomization at the level of single bases. Accounting for this possibility by employing an alternative shuffling method that given a common seed sequence, generated two shuffled versions (one single-shuffled, one dinucleotide-shuffled) of similar sequence divergence (see Materials and methods), yielded the same result. Thus, the smaller variance associated with dinucleotide-shuffling appears to be indeed by a true signal and not an artifact of smaller sequence divergence of dinucleotide-shuffled sequences.

To assess the importance of sequence and composition for cyclizability with regard to single bases and dinucleotides, we compared predicted cyclizability values of actual genomic sequence windows to randomized versions, randomizing either at the level of single bases or dinucleotides. We employed two different randomization strategies, ‘horizontal’ and ‘vertical’ shuffling, see Methods.

**Figure 4.**
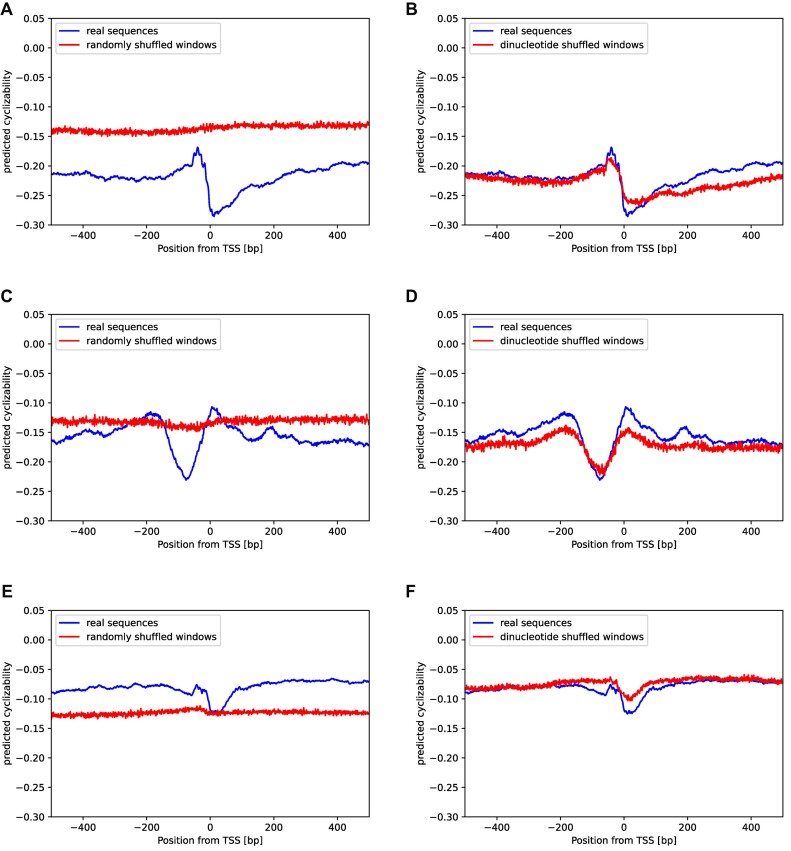
Effects of nucleotide and dinucleotide composition on cyclizability. Mean predicted cyclizability around the TSS of all annotated genes containing a 5′ UTR compared to (left column) shuffled versions of all 50 bp windows of the same sequences, (right column) dinucleotide-shuffled versions of all 50 bp windows of the same sequences in **(A)** and **(B)***Arabidopsis thaliana*, **(C)** and **(D)***Saccharomyces cerevisiae*, **(E)** and **(F)***Homo sapiens*, respectively. Here, the ‘horizontal’ shuffling protocol was applied, i.e. single-bases/ dinucleotides positions were shuffled, unlike drawn from a position-specific probability distribution (‘vertical’ shuffling, Supplementary Figure S1).

When employing the horizontal shuffling protocol, i.e. shuffling sequence windows of length 50 bp horizontally at the level of single bases or dinucleotides), the signal of a characteristic change of cyclizability around TSSs was completely lost (i.e. flat) for single-base-sampling in all three inspected species (*Arabidopsis thaliana*, yeast, and human) (Figure 4A, C, E). By contrast, dinucleotide sampling yielded barely distinguishable cyclizability change profiles compared to the actual profiles (Figure 4B, D, F). However, with regard to absolute level, species differed. While single-base shuffling led to increased values in *Arabidopsis thaliana* and yeast (Figure 4A, C), lowered values were obtained for human (Figure 4E). With dinucleotide shuffling, actual and randomized sequences tracked one another in all three species (Figure 4B, D, F). Applying the vertical shuffling protocol, in which random sequences were generated by statistically sampling from the average composition at specific position near TSSs, either on a single or dinucleotide basis, similar observations were made (Supplementary Figure S1). Mononucleotide-based sampling led to flat profiles, while dinucleotide-based stamping reproduced the profiles associated with actual sequences.

Both shuffling-type experiments indicate that dinucleotide composition plays an important role in explaining characteristic intrinsic cyclizability patterns of genomic DNA around specific genomic features (such as TSSs). This agrees with previous studies that linked dinucleotides as key determinants of certain DNA mechanical properties (30,31,52,53). Of note, at the level of individual sequences (length 50 bp), the correlation of cyclizability values obtained for the actual and dinucleotide-shuffled version was *r* = 0.14 (Pearson correlation coefficient, *r*) only. Yet, when averaged over many sequences, the characteristic profile observed for actual sequences is faithfully reproduced from the dinucleotide-shuffled set (*r* = 0.8).

**Figure 5.**
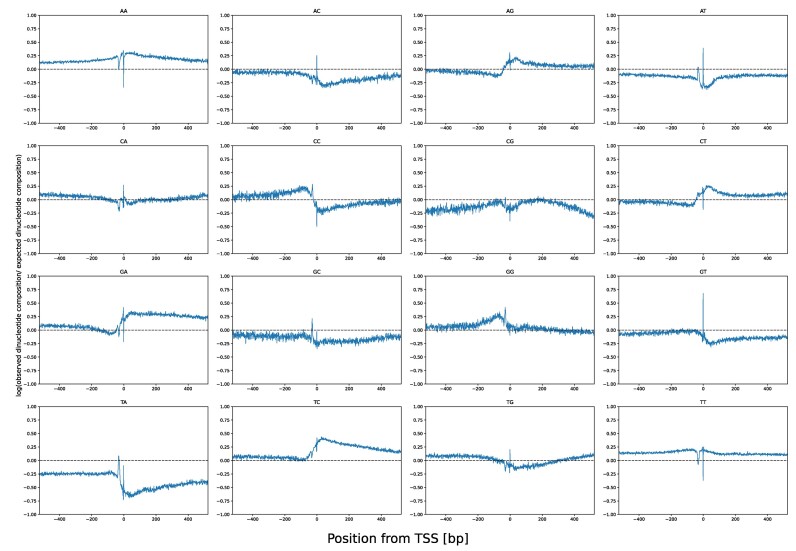
Relative dinucleotide composition around TSSs in *Arabidopsis thaliana*. Natural logarithm of the ratio between the observed dinucleotide composition and the randomly expected dinucleotide composition based on dinucleotide frequency at each position relative to the TSS. Positive/negative log-ratios imply increased/decreased dinucleotide frequencies relative to random expectation, with the dashed line signifying occurrence as expected.

When comparing the cyclizability signal to the distribution of all 16 possible dinucleotides at each position normalized to their random expectation, we found that their relative frequencies change markedly around the TSS and that TA frequency, in particular, correlated strongly with the cyclizability pattern found in *Arabidopsis* (Figure 5). In agreement, the correlation between dinucleotide content and measured cyclizability in the random library reported by Basu *et al.* (28) was found strongest for TA (Pearson correlation coefficient, *r* = 0.19). As the next two strongest correlated (absolute) dinucleotides, TC (*r*= –0.09) and GA (*r*= –0.081) were found negatively correlated with cyclizability, which also can be observed in the dinucleotide composition around the TSS (Figure 5). Similar observations can be made in *S. cerevisiae* (Supplementary Figure S2) and *Homo sapiens* (Supplementary Figure S3).

**Figure 6.**
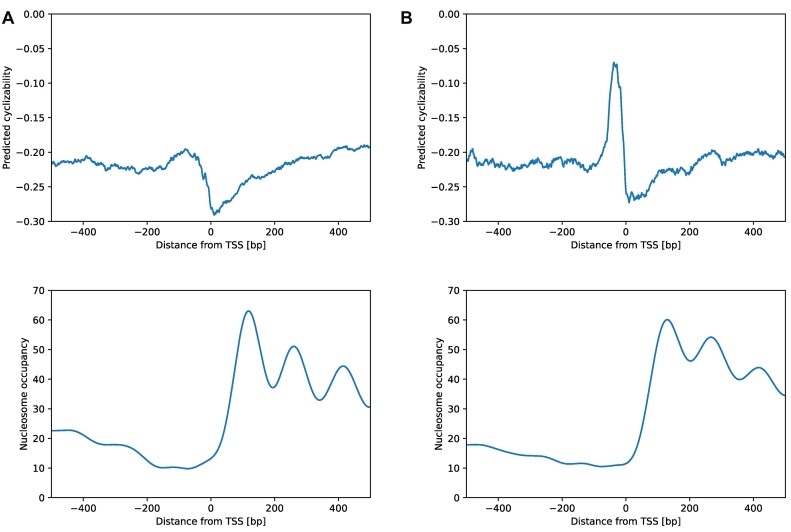
TATA-box genes have a distinct cyclizability pattern. Predicted cyclizability and nucleosome occupancy around transcription start sites (TSSs) of **(A)** genes without TATA-box and **(B)** genes with TATA-box in *Arabidopsis thaliana*.

Two composition peaks, one about 30–40 bp upstream of the TSS and one exactly on the TSS can be observed for most dinucleotides (Figure 5). The latter reflects a high relative occurrence of AT and TG. It seems therefore likely that this peak is largely due to a subset of translation start sites (start codon ATG) being annotated as TSS. GT, which would be part of the alternate start codon GTG/GUG (54), is also found in high frequencies at that position. The TSS-upstream peak coincides with the reported position of the TATA box motif that is frequently found (∼29% (55)) upstream of TSSs in *Arabidopsis thaliana*. The TATA-box is a conserved feature found in many eukaryotic promoters. It is bound by the TATA binding protein (TBP), which recruits the proteins for transcription initiation (56). As the name suggests, the TATA-box consists of a AT/TA rich motif, TATAWAWR. When comparing the cyclizability profile around the TSS of genes containing a TATA-box (*n* = 5369) to genes without TATA-box (*n* = 14 364), we found that TATA-box genes show a pronounced peak of high flexibility upstream of the TSS, which is nearly absent in the other genes (Figure 6A, B). Consistent with that finding, TBPs are known to recognize the high flexibility of its cognate binding motif (57).

It has been reported that plants have a higher number of TATA-box genes compared to vertebrates, which could explain why they show a more pronounced high cyclizability-peak preceding the TSS compared to human or mouse (58). This is consistent with reports from Basu et al. (31), who reported a small flexible peak at the TATA-box position in *S. cerevisiae*.

We then compared the nucleosome occupancy around TSS of TATA-box and TATA-box-less genes. No strong difference could be observed (Figure 6). Both sets show low nucleosome occupancy leading up to the TSS, coinciding with the so-called nucleosome depleted region (NDR), and high occupancy in the gene body, with peaks of high occupancy spaced ∼150 bp apart, which is about the expected size of sequences wrapping around a nucleosome without linker (147 bp) (35). The first peak, representing the +1 nucleosome being the most well defined, consistent with established understanding of nucleosome positioning (11). Interestingly, the reported positive association of cyclizability and nucleosome occupancy (25,59) is evident in the gene body more strongly for TATA-containing genes (Figure 6B) and not (or barely) in TATA-less genes (Figure 6A).

Besides dinucleotide compositions, more complex motifs than dimers can be expected to impact DNA mechanics. Using DeepLIFT (44) importance score, we tried to identify potential motifs with TF-MoDISco (45). However, no motif beyond dimers was identified. Khan *et al.* (30) and using their visible CNN model were able to identify several motifs affecting bendability of a DNA sequence, particularly GAAGAGC and its reverse complement, which was a strong indicator for high flexibility. This effect was confirmed using our own model. Random sequences containing this motif (10 000 sequences, compared to shuffled versions of these sequences) were on average significantly more flexible than dinucleotide-shuffled versions of the same sequence (Cohen's *d* = 2.01, *P* < 0.001).

Taken together, our results indicate that while dinucleotide composition is not sufficient to explain local flexibility at the level of single sequences, it appears to be the dominating factor explaining characteristic flexibility profiles around genomic landmarks such as the TSS and emerging as average-profiles.

### Genomic DNA of many eukaryotes is significantly more rigid than expected by random chance

When comparing the rigidity of sequences close to the TSS to shuffled versions (single-base level) of the same sequence in Arabidopsis, we noticed that the original sequences were, on average, more rigid than their randomized versions. Similarly, when we compared predicted cyclizability around the TSS of different species, the investigated vertebrates (mouse and human) showed a consistently higher flexibility (Figure 2I). A similar observation was made by Li *et al.* (29), who reported that mammals and thermophilic archaea had, in general, a more flexible genome than yeast, *E. coli* and T4 Phage.

**Figure 7.**
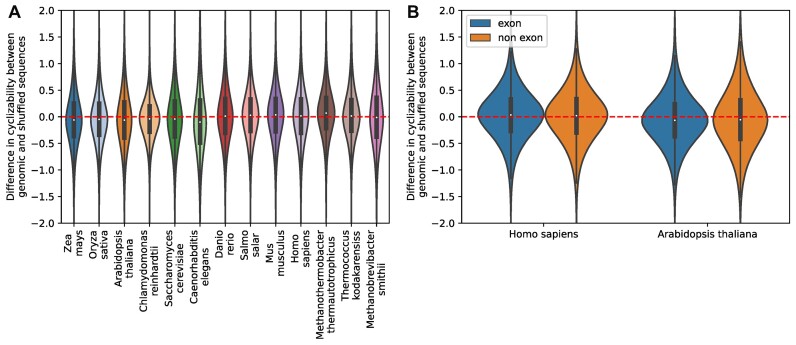
Mean genomic cyclizability of different species relative to randomized versions of the same sequence. **(A)** Distribution of differences between predicted cyclizability of genomic sequences and randomly shuffled versions of the same sequences for different eukaryotes and three archaea (Mth, Tko, and Msm) species. The individual values were derived by predictions of cyclizability of 50 bp windows at 50 bp step-size (spacing between windows) from the respective genomes, randomly shuffling those sequences five times, and then calculating the difference, *D*, in cyclizability between shuffled and original sequence with *D* = C_a_– C_r_, C = cyclizability, a = actual, r = randomized. All individual differences are represented by their frequency distributions. Positive values indicate genomic sequences to be more flexible than their randomized versions, negative values the opposite. **(B)** Distribution of differences between predicted cyclizability of exon/non-exon sequences and randomly shuffled versions of the same sequences for *Homo sapiens* and *Arabidopsis thaliana*. Values were calculated as described under (A).

We tested whether these observations are statistically significant, and if the genomes of the respective organisms are generally more flexible or rigid than expected by chance. We tiled genomic sequences of each organism into non-overlapping windows of length 50 bp, and compared their predicted cyclizability to predicted cyclizability values of the same sequences randomly shuffled (single-base shuffling, five repeats). Thus, we considered a locally shuffled genomic sequence as a ‘random genome’. This preserves locally the base composition, while keeping larger compositional differences across longer genomic regions intact. This revealed that in all eukaryotic organisms investigated, except vertebrates, genomic sequences were significantly more rigid than the randomly shuffled sequences (t-test, *P* << 0.0001, please see comment on *P*-values at the end of this paragraph) (Figure 7A). In all non-vertebrate eukaryotes, the effect size between original and randomized sequences ranged from 0.183 in *C. elegans* to 0.101 in yeast. In the case of vertebrates, the opposite was true, with all of them being, on average, more flexible than randomly shuffled sequences, however with considerably smaller effect sizes, ranging from 0.08 in salmon (*Salmo salar*) to 0.029 for human. When comparing the distributions of differences between genomic vs. shuffled, the largest interspecies difference was found between salmon and *C. elegans*, with an effect size of 0.3, the smallest between zebrafish and mouse, with an effect size of 0.0026.

To exclude composition-based effects, we compared the randomly shuffled sequences to each other. While all differences were significant (*P* << 0.001), the effect sizes were small, with the largest difference being between *Chlamydomonas reinhardtii* and *C. elegans* with an effect size of 0.047 and the smallest between maize and rice, with effect size smaller than 0.001. Chlamydomonas was generally an outlier, with the highest mean cyclizability of all shuffled sequences. This is most likely an effect of its very high GC content of Chlamydomonas of around 64% (60) compared to the other eukaryote species (∼35–40%). Excluding Chlamydomonas, the highest effect size between randomly shuffled sequences was 0.031. Therefore, it seems likely that the difference in mean cyclizability between genomes is an effect that is unrelated to genome-specific sequence composition.

The occurrence of repetitive DNA-elements could be another possible explanation for differences between species. Vertebrates are known for their large genomes, containing high percentages of repetitive sequences, while most chosen non-vertebrate organisms have relatively short, gene-dense genomes (61). As a test for a potential effect, we compared sequences lying in exons to non-exon sequences in *Arabidopsis* and human, respectively, following the rationale that exons are generally devoid of repetitive sequences. While the respective differences were significant (*P* < 0.001) due to the large sample size, the very small effect sizes of 0.015 and 0.013 indicated that there were no relevant differences in either species (Figure 7B).

When analyzing three selected archaea species, two thermophiles also investigated by Li *et al.* (29), *Methanothermobacter thermautotrophicus* and *Thermococcus kodakarensiss*, and the mammalian commensal *Methanobrevibacter smithii*, we found a strong difference between the species, similar to what we observed in eukaryotes. The two thermophiles (*M. thermautotrophicus* and *T. kodakarensiss*) were significantly more flexible than expected (*P* << 0.001), with effect sizes of 0.21 and 0.11, respectively. *Methanobrevibacter smithii* however was significantly less flexible than expected (*P* < 0.05), albeit with a small effect size of 0.013.

Please note, with regard to the reported *t*-test *P*-values reported in this paragraph, we wish to point out that the *P*-values are heavily influenced by the large number of observations, *N* (millions of prediction windows and the five shuffles) allowing even small differences of mean values to be significant. Hence, the effect sizes should be taken as the more informative measure, as it compares the difference of mean values to the averaged standard deviations, which are not influenced by *N*, and thus are informative with regard to the magnitude of difference. Roughly, an effect size of 0.1 suggests that the difference of means is 10% of the average standard deviation.

### A subgroup of *Arabidopsis thaliana* transcription factors shows distinct DNA rigidity patterns at their target site when observed occupied compared to non-occupied locations of the same motif

The binding of transcription factors (TFs) to DNA is one of the most investigated instances of the role of DNA mechanics. Of particular interest has been the observation that some instances of a TF binding site motif are bound by the respective TF, while for other instances of the same motif, the respective TF shows a much lower affinity. One possible reason could be the local flexibility of DNA, promoting or inhibiting protein binding. Local flexibility is not only dependent on the sequence of the motif, but also the flanking regions. The flanking regions of motifs, while less conserved, play an important role in TF motif recognition and binding (62). Recently, this has been linked to the shape and flexibility of these regions (17,18).

**Figure 8.**
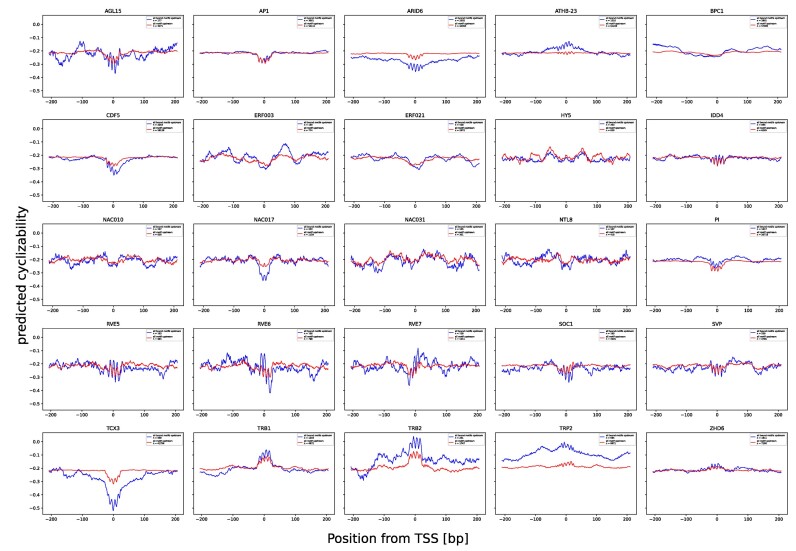
Comparison of bound and unbound instances of transcription factor binding site motifs in gene promoter regions in *Arabidopsis thaliana*. Mean predicted cyclizability of all bound occurrences of transcription factor binding site motifs of a given transcription factor with FIMO *q*-value score <0.1 (blue profiles) versus all occurrences of the binding motif with the same confidence (red profiles), centered around the binding motif. Only occurrences in 1 kb upstream of annotated TSS were considered in the analysis. TF-binding (bound versus unbound) was taken as evidenced by DAP-seq support. Presented are all TFs, which met the following criteria: at least 100 found instances of FIMO hits with a *q*-value below the 0.1 threshold in regions up to 1000 bp upstream of any TSS.

To investigate this further, we used available DAP-seq data for Arabidopsis TFs (41) and their reported motifs, represented by their position weight matrices (PWM) available on JASPAR, to find bound (or occupied) and unbound (non-occupied) motif occurrences in the region 1kbp upstream of any transcription start site (TSS). DAP-seq data were taken as evidence of TF-binding, while absence thereof at respective motif locations were taken as an indication of no binding. The opposite can be observed when looking at TRP2, which showed a flexible binding site. In this analysis, in order to investigate whether the mechanical properties of the bound regions are actually promoting TF binding, or are only a byproduct of the specific binding sequence, we compared bound motifs to unbound motifs with a *q*-value <0.1 identified by the program FIMO. For each motif, all bound instances including their –200 and +200 bp flanking region were selected, and its cyclizability profile was predicted. Inspecting the 25 TFs, for which sufficient data was available (Figure 8), we observed that while the majority of TFs showed no clear signal at their binding site with detected binding, others (seven, based on visual inspection: ARID6, ATHB-23, CDF5, NAC017, TCX3, TRB2 and TRp2) have a distinct pattern of predicted rigidity at their binding motif relative to all motif instances. For example, TCX3 shows a distinct pattern of strong negative cyclizability, indicating a preference for rigid binding sites.

Taken together, this indicates that the significance of mechanical properties around the binding site may depend on the specific TF.

### Sites that can undergo methylation in *Arabidopsis thaliana* show no preference in local cyclizability

**Figure 9.**
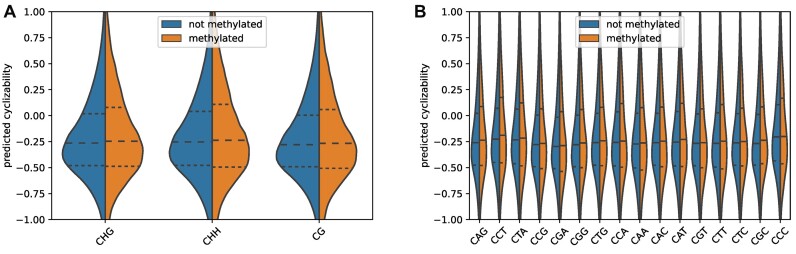
Comparison of cyclizability between methylated and non-methylated positions in *Arabidopsis thaliana*. Comparison of the distributions of predicted cyclizability of methylated and unmethylated sites in the same methylation sequence context in promoter regions (300 bp upstream of the TSSs) of *Arabidopsis thaliana*. Comparison of **(A)** the three main methylation contexts as represented by their consensus motifs: CHG, CHH and CG, **(B)** all specific methylation contexts. Horizontal dashed lines denote 25, 50 and 75 percentiles, respectively. Note that it is not the effect of methylation that is examined, but whether a cytosine with a particular sequence context was observed to undergo methylation.

DNA methylation is an epigenetic modification, in which the C5 position of a cytosine is modified by addition of a methyl group. It regulates a wide variety of functions, including gene expression and chromatin regulation. In plants, it can appear in three different sequence contexts, CG, CHG and CHH. DNA-methylation, in turn, has also been shown to decrease DNA flexibility (31,63). Since Basu *et al.* were able to show that certain chromatin remodelers were sensitive to local DNA flexibility (28), it is reasonable to hypothesize that establishing DNA methylation might also be influenced by local mechanical DNA properties, such that DNA methyltransferases may have a preference for either low or high local DNA flexibility. Using the methylation calls for *Arabidopsis thaliana* (accession, Col-0) from the 1001 epigenome project (37), we predicted the cyclizability of all sequence windows containing C-positions that have been observed to undergo methylation and compared them to random sequence windows with the same consensus context (CG, CHG or CHH), but no reported methylation. To eliminate differences in overall sequence composition and functional relevance as a possible confounding factor, we confined this comparison to sequence windows falling into promoter regions. Sequence windows with methylated positions had a slightly higher cyclizability value in all three sequence contexts than sequence windows containing the same sequence context but without evidence of being methylated. However, the effect sizes were small (*d* = 0.039, 0.059, 0.053, respectively), indicating that these effects may not be biologically relevant (Figure 9A). When inspecting all individual sequence contexts covered under the CHG and CHH motif descriptions, the effects were still small, however, some contexts such as CCT, CGC and CAT had slightly larger effect sizes of 0.102, 0.084 and 0.075 (Figure 9B).

### *De novo* mutations and fixed SNPs in *Arabidopsis thaliana* appear more frequently in rigid genomic DNA contexts

A recent paper by Monroe et al. revealed that mutations, unlike previously assumed, do not occur at random, but are themselves biased reflecting natural selection (39). Mutations, accruing *de novo* in the experimental series, were found to occur less often in functional constrained regions of the genome, i.e. gene bodies. This suggests that DNA modifications, DNA accessibility as well as intrinsic properties of certain functional genomic regions could influence the likeliness of *de novo* mutations. They reported that the region upstream of the TSS showed significantly higher mutation rate than the gene body. The mutation rate profile around TSSs behaved very similarly to the predicted cyclizability observed in this study (39).

This led us to surmise that DNA flexibility might be a factor influencing the *de novo* mutation rate. Using the provided data from Monroe et al., we examined all sequence windows containing an observed de novo mutation and compared them to all sequence windows with no reported *de novo* mutation. Again, to exclude any composition and position related effects, we focused on gene promoters. Considering all sequence windows up to 300 bp upstream of a TSS, windows containing *de novo* mutations were significantly more rigid than windows not containing *de novo* mutations (*P* < 0.001), with an effect size of 0.161. When comparing all windows (i.e. not only near TSSs) with and without *de novo* mutations on Arabidopsis chromosome 1, i.e. without focusing on any specific functional genomic region, the same trend was observed, with an effect size of 0.134. This implies that rigid regions are generally more susceptible to *de novo* mutations.

**Figure 10.**
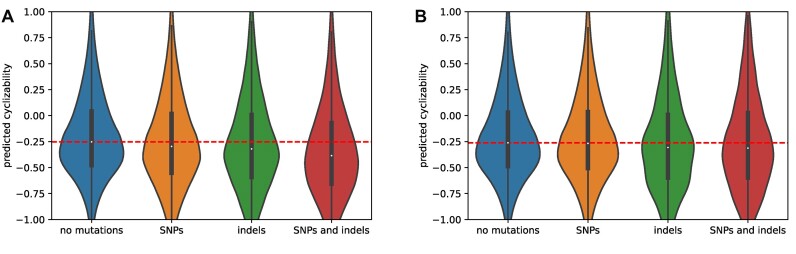
Cyclizability of genomic sequence windows with and without variants in *Arabidopsis thaliana*. **(A)** Comparison of predicted cyclizability between sequences containing no *de novo* mutation, SNPs only, indels only, and both SNP and indel *de novo* mutations. **(B)** Comparison of predicted cyclizability between sequences containing no fixed mutations (fixed = occurring in naturally occurring accessions, SNPs only, indels only, and both SNP and indel mutations according to the 1001 genome project. The dashed lines visualize the median cyclizability of the respective ‘no mutation’ windows as a reference.

Next, we grouped sequence windows into four classes based on presence/absence of particular *de novo* sequence variants—no mutation, only single nucleotide polymorphism (SNP) mutation, only indel (insertions/deletions) mutation and SNP and indel mutation. Compared to sequence windows with no mutations, sequence windows of all three mutation classes had lower cyclizability, and are therefore predicted to be less flexible, consistent with the results reported above. However, the strength of the effect varied among the groups (Figure 10A). In promoter regions, sequence windows with only SNPs had the lowest effect size of 0.121 when compared to windows without mutations. Windows containing only indel mutations had an effect size of 0.182. Lastly, the strongest effect could be observed when comparing windows containing both SNP and indel mutations, with an effect size of 0.334. When performing the same experiments on all windows on chromosome 1, we found similar results, with effect sizes of 0.106, 0.143 and 0.258 for only SNP, only indel and both SNP and indel mutations, respectively.

We then investigated whether this effect can also be observed for fixed SNPs, i.e. naturally occurring SNPs, and short indels, as reported by the 1001 genome project (38), again restricting the analysis to 300 bp promoter regions to avoid potential differences of conservation due to a different functional relevance of particular genomic regions. When comparing all sequence windows containing mutations to all windows without mutations, we found that SNP-containing windows were significantly more rigid than non-SNP windows, as observed with *de novo* mutations. The effect size was smaller (0.023) compared to the *de novo* mutation effect sizes, potentially indicating that there is no relevant, or a lesser contribution of mechanical factors in fixed than in *de novo* variants. The different mutation types (SNP, indel, SNP + indel) behave similarly as observed for *de novo* mutations, with effect sizes of 0.022 for SNPs, 0.125 for indels and 0.124 for SNPs and indels relative to sequence windows devoid of mutations.

### Sites of high Spo11 oligonucleotide accumulation in *Arabidopsis thaliana* showed no correlation with DNA flexibility

During meiosis, crossing-over is initiated by double-strand breaks and subsequent DNA repair. These double strand breaks are introduced by Spo11, a relative of topoisomerases (64). Since it has been shown that Spo11-induced breaks are associated with nucleosome depleted regions (65), we suspected that Spo11 might have an affinity for rigid DNA. Using the Spo-11 oligonucleotide mapping in Arabidopsis from Choi *et al.* (42), we compared those values to the cyclizability values for sequence windows of length 50 bp centered on the same position. Again, we focused on promoter regions (1000 bp upstream of TSSs) to study a particular genomic region, thereby reducing the effect of confounding factors, notably nucleosome occupancy as upstream regions tend to be depleted of nucleosomes. Spo11-oligonucleotides and cyclizability showed no relevant correlation (*r* = 0.002), and hence no evidence for an association of DNA flexibility and double-strand breaks was found.

## Discussion

The investigation of mechanical properties of genomic DNA and their possible functional implications has a long research tradition. The recent development of novel experimental techniques that allow probing for mechanical properties at a whole-genome scale, such as loop-seq (28), has spawned renewed interest as it allowed addressing possible functional consequences of mechanical properties of DNA at an unprecedented scale (31). The generated loop-seq dataset also perfectly lent itself to the development of machine learning methods that predict mechanical bendability (cyclizability) from sequence, allowing to expand the analysis scope beyond the examination of actual experimental data, but to apply the respectively developed algorithms to other species and a broad range of genomic contexts (29–31). As a main premise of such generalization, the assumption has to be made that the sequence-structure relationships of genomic DNA are universal, an assumption that seems plausible. Following this rationale, we aimed to expand the analysis to a range of particular associations between mechanical properties and functional sites in genomes not addressed previously For this, we developed, CycPred, a computational artificial neural network prediction model on the basis of available experimental data of genomic sequence cyclizability (28). CycPred was observed to perform at high accuracy levels, fast, and stably across diverse input sequence sets. As part of their study, Basu et al. also developed a linear prediction model that incorporates deliberately chosen features such as dinucleotide pair frequencies and their distances, yielding correlation levels of predicted versus observed data of around *r* ∼0.51–0.55 (31). While a model with interpretable, engineered features allows for direct interpretation, the neural network architecture employed here proves more accurate, though posing challenges with regard to interpretation. Applying CycPred, we systematically examined how different aspects and characteristic loci of eukaryotic genomes may be affected by DNA flexibility.

Observed for several and diverse species, transcription start sites seem to be associated with pronounced changes of local DNA flexibility (Figure 2). It needs to be asked whether this characteristic profile is itself functionally relevant or a consequence of an overrepresentation of particular sequence motifs signifying TSSs and for reasons other than mechanical properties. In particular, the region immediately upstream of TSSs, the core-promoter, is known to harbor characteristic motifs (such as frequent occurrences of TATA-boxes) and, furthermore, in Arabidopsis was shown to exhibit characteristic dinucleotide orientational preferences (66). Thus, the observed profile of mean cyclizability near TSSs may result for those motifs, and whether the associated flexibility itself is of functional relevance will have to be determined. As an indication of the direct significance of flexibility, Basu *et al.* showed that chromatin remodelers (INO80) may sense DNA mechanical properties near TSSs (28).

We observed that the average TSS-cyclizability profile is determined almost entirely by dinucleotide composition (Figure 4, Supplementary Figure S1). Similarly, dinucleotide composition has been found correlated with the specific cyclizability profiles at nucleosome positions (29). The important role of dinucleotides has also been illuminated by Basu *et al.* (31). Training a linear model on dinucleotide frequencies and their pairwise sequence-distance distributions, predictions of cyclizability values at the level of individual sequences was possible at a correlation level of ∼0.55 (Pearson correlation coefficient). Our results seem to suggest a possibly minor significance of pairwise distances of dinucleotides when averaged over many sequences aligned on TSSs. Horizontally shuffling the sequences at the dinucleotides, i.e. preserving composition, but destroying pairwise distances, resulted in a near faithful reproduction of average cyclizability profiles around TSSs (Figure 4). Yet, while, on average, the profiles obtained for actual and dinucleotide-randomized sequence versions were very similar (*r* = 0.8), their actual mean correlation over pairwise (shuffled vs. actual) windows was detected at *r* = 0.14 only, i.e. individually, substantial scatter is present. Also, at the level of individual sequences, local variance of cyclizability along the sequence seems very high as indicated by the rapid decay of the autocorrelation function (Figure 3). Thus, any importance of DNA flexibility may become apparent only when averaging over many sequences (aligning on the TSS, for example). While then, a clear characteristic profile is evident (Figure 2), it can be reproduced based on dinucleotide composition alone, irrespective of pairwise sequence separation. We conclude that while at the level of individual sequences, pairwise distances may play a role (as convincingly shown by Basu *et al.* (31)), for their importance as a characteristic around genomic landmarks, local composition alone may be the dominating feature.

Particular dinucleotides have already been reported to impart special mechanical properties. For example, TA, frequently referred to as a pyrimidine–purine step, has been implied to be associated with high flexibility (26,31) and to introduce local kinks (67), possibly involving a switch of base pairing from ‘Watson–Crick’ to ‘Hoogsteen’ (68). Given the TATA-box (literally containing the core motif sequence TATA), the high flexibility observed for regions upstream of the TSS may be explainable.

Here, we focused on dinucleotides as the next higher level of k-mers relative to mononucleotides and observed that already at this first complexity increment, average cyclizability profiles are captured much better than obtained with mononucleotides (Figure 4). A search for higher-order motifs performed in this study did not identify motifs, except confirming the reported GAAGAGC motif and its reverse complement (30). Evidently, when referring to ‘mono’ or ‘di’-nucleotides, their respective actual canonical base-pairs of length one or two are meant.

In the seminal paper on the experimentally determined DNA-cyclizability in yeast (28), which forms the basis of this study, a region of lowered flexibility upstream of TSSs has been reported in yeast, and confirmed here using our computational prediction method (Figure 2E). This region was reported as a stiff region, in which nucleosome binding/formation is impaired, thereby facilitating TF-binding. Surprisingly, this pattern was not observed in any of the other species examined here (Figure 2). Rather, we observed lowered cyclizability right on or immediately downstream of the TSS. Assuming the prediction method as well as the TSS annotation to be reliable, this may either point to the yeast genome to be special, or that the stiffness of the TSS itself may be relevant for transcription initiation.

Interestingly, several investigated eukaryotic genomes, but not vertebrates, were significantly more rigid compared to randomized sequences (Figure 7A). This effect was largely base-composition-independent. The significance of this observation is not yet clear. It could serve some functional reason, or be the result of some underlying factor unrelated to rigidity, like repetitive sequences. However, there were only minimal differences between exon and non-exon sequences in both Arabidopsis or human (Figure 7B), indicating that repetitive sequences may not be the reason. Of note, as there many conceivable ways to randomize genomic sequences, here we always performed the comparison of actual to randomized sequences locally, averaged over the whole genome under study.

While we observed that actual genomic sequences are often more rigid than their randomized versions, with cyclizability taken as a surrogate of flexibility, DNA as a whole still appears to be mechanically very flexible, as was discussed before (23–25). Interestingly, the ∼50 bp autocorrelation decay distance of cyclizability (Figure 3) is even shorter than the reported persistence length of DNA, around 150 bp (69). The cyclization of short DNA sequences (25) can thus not be explained with the standard worm-like chain model from which the persistence length was derived (23). However, to explain many functions of DNA, such as TF binding or other protein DNA interactions, a model allowing higher flexibility of DNA may indeed be biologically very important, and thus, the measured cyclizabilities real and relevant. Several explanations for the observed discrepancy, like the formation of single-stranded bubbles (70), or kinks (71,72) have been suggested.

Using autocorrelation as an indicator, no long-range persistence of predicted cyclizability was found (Figure 3A, B). However, this conclusion does not contradict earlier findings that cyclizability exhibits a periodic behavior related to periodic nucleosome positions (29,73), and found here in *Arabidopsis thaliana* as well (Figure 6B), but indicates that any systematic trends with regard to flexibility as measured by local cyclizability are revealed only, when multiple instances of alignable sequences are averaged, with alignable meaning that sequences can be positioned relative to specific landmark sign-posts, e.g. dyads or transcription start sites. Then, and as shown before (28,29,31), averaged over many sequences, systematic trends of local flexibility can be revealed. But at any given individual site, local variability of cyclizability will be very high, in fact, higher than the signal discerned from averaging.

Temperature is a known evolutionary factor shaping genome composition and structure. Thermophilic bacteria and archaea have been reported to have high GC content, which has been linked to thermal adaptation due to higher thermostability and potentially increased DNA repair efficiency (74). Li *et al.* (29) reported high average cyclizability, and thus high mechanical flexibility, for *Methanothermobacter thermautotrophicus* and *Thermococcus kodakarensiss*, two thermophilic archaea. This can partly be explained with their high GC content of 50% and 52% respectively, since long poly(dA:dT) stretches have been found associated with rigid DNA sections (29,75,76). When we compared genomic sequences of those two archaea to randomly shuffled versions of these sequences, we could show that their genome was significantly more flexible than expected by random chance (Figure 7A). However, Chlamydomonas, with an even higher GC content of 64%, has a significantly lower median cyclizability and is more rigid than expected by random chance. Li et al. suggested that the nucleosome structure of thermophile archaea species, wrapping only 60 bp around nucleosomes (77), requires their genomes to be more flexible. In contrast to the thermophiles with high flexibility, we found that the genome of *Methanobrevibacter smithii*, an archaea commensal, was observed to be overall less flexible (Supplementary Figure S4), and showed no strong difference compared with randomized sequences (Figure 7A). Assuming that flexible DNA is required for the tight nucleosome wrapping observed in *T. kodakarensiss* and *M. thermautotrophicus*, it is less likely to find this nucleosome organization in *M. smithii*. This may reflect the large variability of genome organization found in archaea compared to eukaryotes (78).

Investigations of transcription factor binding showed a distinct preference for rigid/flexible areas of DNA for some transcription factors (Figure 8). By comparing bound and unbound motifs, we could show that some TFs had a more pronounced flexibility motif in bound instances. Since transcription factor binding site motifs are relatively short sequences (∼6–15 bp), the flanking regions have a large impact on the flexibility profile. This agrees with previous reports that the sequence of TF motif flanking regions, while not forming distinct motif themselves, have a large impact on TF binding specificity. Specifically, DNA-shape and flexibility of the flanking regions have been suggested as major contributors to this effect (18–20,79). It would be interesting to further investigate, which TFs show these preferences, and explore, which mechanism is potentially responsible.

DNA modification by methylation is a key feature of eukaryotic genomes. Our investigation found no strong connection between rigidity surrounding cytosines (in CG, CHG, CHH sequence context, respectively) and whether or not they have been observed to be methylated. This implies that the methyltransferases establishing DNA methylation show no particular dependence on the flexibility of DNA, at least not in the plant *Arabidopsis thaliana* (Figure 9). Please note, we did not test for the effect of methylation on DNA flexibility, but whether the target sites of DNA methylation themselves show characteristic DNA flexibilities. We applied a model that was trained on unmethylated data. With regard to the effect of an actual DNA-methylation on the flexibility of genomic DNA, Basu *et al.* (31) reported that generally, CpG-methylation leads to a stiffening of DNA.

By contrast, *de novo* mutations as reported by Monroe *et al.* (39) have been found located in rigid sequence contexts. Specifically, sequence windows containing both SNP and indel mutations were found with increased rigidity (Figure 10). This effect is also observable, albeit less pronounced, in fixed SNPs and indels from the 1001 genome project. This effect could help to explain the different mutation rates observed among different species (80), as we observed different median cyclizability values in genomes of different species (Figure 7A). For naturally occurring polymorphisms in *Arabidopsis thaliana*, which we found associated with slightly stiffer sequences, a sequence window that contains SNPs will correspond to different genotypes (actual sequences) in different Arabidopsis accessions, and thus different cyclizabilities. However, it is extremely unlikely that the representative accession used here (Col-0) always corresponds to the stiffer of the various occurring sequences in the different accessions.

It has been previously reported that DNA bound to nucleosomes accumulate more SNP mutations due to an inhibition of certain DNA repair pathways, while linkers showed a higher indel mutation rate. In this context, mechanical properties may play a role as linkers have been reported by previous studies to be more rigid (28,29). Non-homologous end joining (NHEJ) repair of double-strand breaks has been associated with indel mutations (81,82). It is therefore possible that rigid DNA sections are more susceptible to lesions resulting in double-strand breaks, or more prone to errors during DNA repair. Topoisomerases are enzymes that introduce single- and double-strand breaks into DNA. Their main function is reduction of torsion by reducing positive/negative supercoiling, for example during transcription, which itself could be linked to local DNA mechanics. Therefore, if topoisomerases show a preference for certain local DNA mechanical properties, a higher mutation rate at those positions due to more frequent DNA double strand breaks could be a possible explanation. There have been reports that Topoisomerase 1 is associated with transcription dependent mutagenesis (83,84). However, our investigation of Spo11, a relative of topoisomerase, yielded no indication of a preference for rigid regions of DNA.

With regard to technical aspects, we developed a computational prediction model, and reported results obtained by applying it, based on experimental data from yeast. For the original data, our prediction accuracy proved very high (*r* = 0.93, with regard to true versus predicted cyclizability obtained for the reported ‘random sequence set’, *r* = 0.77 for chromosome V). We then applied the model to other species, i.e. different genomic sequences. In as much as (i) DNA is universally the same polymer following the same sequence-structure relationships, (ii) the experimental data were obtained for ‘naked’ and synthetic DNA, i.e. without chemical modifications or bound proteins that could be species-specific and (iii) the model was trained on very large sequence datasets, and showed high performance on a random library thus we believe a transfer to other species is plausible and will likely generate valid predictions even if the sequences will differ.

Our results are based on statistical associations. Experimental evidence is needed to confirm them as association does not imply causation, and other sequence constraints could partly explain the reported observations.

Furthermore, our results rely on the genomic sequence and annotations to be correct. In particular with regard to the position of transcription start sites, this is, for principle—given alternative start sites—and technical reasons, challenging. However, we believe that the fact that we did observe characteristic profiles around TSSs in all species examined (Figures 2, 5, Supplementary Figures S2 and S3), even in view of potential sources of error, suggests that indeed a signal is present.

## Conclusions

Our study explored a range of sequence-structure-function relationships of genomic DNA that may be influenced by local DNA flexibility. We found a characteristic change of flexibility near transcription start sites that was found consistently across multiple species and largely driven by dinucleotide compositional effects. Unlike previously discussed as an indication of facilitated access of transcription factors to gene promoters, no evidence of a generally present region of lowered flexibility upstream of transcription start sites was found. Yet, with regard to transcription factor binding, depending on the actual transcription factor, flanking-sequence-dependent flexibility was observed as a potentially important factor influencing binding. Compared to randomized genomic sequences, depending on species and taxa, actual genomic sequences were globally observed both with increased and lowered flexibility. Lastly, our analysis suggests that mutation rates may be linked to local DNA mechanical properties, with rigid areas being more susceptible to SNPs (*de novo* and fixed) and, in particular, indel mutations. Sites of double-strand breaks, however, show no evidence for being associated to DNA-flexibility.

Taken together, our study presents a range of significant correlations between characteristic DNA mechanical properties and genomic features, the significance of which with regard to detailed molecular relevance awaits further experimental and theoretical exploration.

## Supplementary Material

lqad097_Supplemental_File

## Data Availability

CycPred is available at https://figshare.com/articles/software/CycPred/24315502 (DOI 10.6084/m9.figshare.24315502) and https://github.com/georgback/CycPred.

## Note added in proof

During the proof-reading stage we became aware of a new publication reporting also on the development of an artificial neural network architecture for the prediction of DNA cyclizability, using the same input and reporting a similar performance as obtained by us, and presenting a study on the potential relevance of DNA bendability (85).

## References

[1] Lamm E. , Harman O., Veigl S.J. Before Watson and Crick in 1953 came Friedrich Miescher in 1869. Genetics. 2020; 215:291–296. 32487691 10.1534/genetics.120.303195 PMC7268995

[2] Brázda V. , Laister R.C., Jagelská E.B., Arrowsmith C. Cruciform structures are a common DNA feature important for regulating biological processes. BMC Mol. Biol. 2011; 12:33. 21816114 10.1186/1471-2199-12-33 PMC3176155

[3] Yang D. Yang D. , Lin C. G-Quadruplex DNA and RNA. G-Quadruplex Nucleic Acids: Methods and Protocols. 2019; NY Springer 1–24.Methods in Molecular Biology.

[4] Ravichandran S. , Subramani V.K., Kim K.K. Z-DNA in the genome: from structure to disease. Biophys. Rev. 2019; 11:383–387. 31119604 10.1007/s12551-019-00534-1 PMC6557933

[5] Whelan D.R. , Hiscox T.J., Rood J.I., Bambery K.R., McNaughton D., Wood B.R. Detection of an en masse and reversible B- to A-DNA conformational transition in prokaryotes in response to desiccation. J. R. Soc. Interface. 2014; 11:20140454. 24898023 10.1098/rsif.2014.0454 PMC4208382

[6] Jiang C. , Pugh B.F. Nucleosome positioning and gene regulation: advances through genomics. Nat. Rev. Genet. 2009; 10:161–172. 19204718 10.1038/nrg2522 PMC4860946

[7] Ngo T.T.M. , Zhang Q., Zhou R., Yodh J.G., Ha T. Asymmetric unwrapping of nucleosomes under tension directed by DNA local flexibility. Cell. 2015; 160:1135–1144. 25768909 10.1016/j.cell.2015.02.001 PMC4409768

[8] Peckham H.E. , Thurman R.E., Fu Y., Stamatoyannopoulos J.A., Noble W.S., Struhl K., Weng Z. Nucleosome positioning signals in genomic DNA. Genome Res. 2007; 17:1170–1177. 17620451 10.1101/gr.6101007 PMC1933512

[9] Suter B. , Schnappauf G., Thoma F. Poly(dA·dT) sequences exist as rigid DNA structures in nucleosome-free yeast promoters in vivo. Nucleic Acids Res. 2000; 28:4083–4089. 11058103 10.1093/nar/28.21.4083 PMC113125

[10] Segal E. , Widom J. Poly(dA:dT) tracts: major determinants of nucleosome organization. Curr. Opin. Struct. Biol. 2009; 19:65–71. 19208466 10.1016/j.sbi.2009.01.004 PMC2673466

[11] Radman-Livaja M. , Rando O.J. Nucleosome positioning: how is it established, and why does it matter?. Dev. Biol. 2010; 339:258–266. 19527704 10.1016/j.ydbio.2009.06.012 PMC2830277

[12] Saran R. , Wang Y., Li I.T.S. Mechanical flexibility of DNA: a quintessential tool for DNA nanotechnology. Sensors. 2020; 20:7019. 33302459 10.3390/s20247019 PMC7764255

[13] Wu H.-M. , Crothers D.M. The locus of sequence-directed and protein-induced DNA bending. Nature. 1984; 308:509–513. 6323997 10.1038/308509a0

[14] Vámosi G. , Rueda D DNA bends the knee to transcription factors. Biophys. J. 2018; 114:2253–2254. 29229184 10.1016/j.bpj.2017.10.047 PMC6129554

[15] Huber E.M. , Scharf D.H., Hortschansky P., Groll M., Brakhage A.A. DNA minor groove sensing and widening by the CCAAT-binding complex. Structure. 2012; 20:1757–1768. 22902862 10.1016/j.str.2012.07.012

[16] Harteis S. , Schneider S. Making the bend: DNA tertiary structure and protein-DNA interactions. Int. J. Mol. Sci. 2014; 15:12335–12363. 25026169 10.3390/ijms150712335 PMC4139847

[17] Sielemann J. , Wulf D., Schmidt R., Bräutigam A. Local DNA shape is a general principle of transcription factor binding specificity in Arabidopsis thaliana. Nat. Commun. 2021; 12:6549. 34772949 10.1038/s41467-021-26819-2 PMC8590021

[18] Yella V.R. , Bhimsaria D., Ghoshdastidar D., Rodríguez-Martínez J.A., Ansari A.Z., Bansal M. Flexibility and structure of flanking DNA impact transcription factor affinity for its core motif. Nucleic Acids Res. 2018; 46:11883–11897. 30395339 10.1093/nar/gky1057 PMC6294565

[19] Chiu T.-P. , Li J., Jiang Y., Rohs R. It is in the flanks: conformational flexibility of transcription factor binding sites. Biophys. J. 2022; 121:3765–3767. 36182667 10.1016/j.bpj.2022.09.020 PMC9674972

[20] Ghoshdastidar D. , Bansal M. Flexibility of flanking DNA is a key determinant of transcription factor affinity for the core motif. Biophys. J. 2022; 121:3987–4000. 35978548 10.1016/j.bpj.2022.08.015 PMC9674967

[21] Travers A.A. The structural basis of DNA flexibility. Philos. Trans. R. Soc. Lond. Ser. Math. Phys. Eng. Sci. 2004; 362:1423–1438. 10.1098/rsta.2004.1390 15306459

[22] Mills J.B. , Hagerman P.J. Origin of the intrinsic rigidity of DNA. Nucleic Acids Res. 2004; 32:4055–4059. 15289578 10.1093/nar/gkh740 PMC506819

[23] Du Q. , Smith C., Shiffeldrim N., Vologodskaia M., Vologodskii A. Cyclization of short DNA fragments and bending fluctuations of the double helix. Proc. Natl. Acad. Sci. 2005; 102:5397–5402. 15809441 10.1073/pnas.0500983102 PMC556251

[24] Vafabakhsh R. , Ha T. Extreme bendability of sub-100 bp long DNA revealed by single molecule cyclization. Science. 2012; 337:1097–1101. 22936778 10.1126/science.1224139 PMC3565842

[25] Cloutier T.E. , Widom J. Spontaneous sharp bending of double-stranded DNA. Mol. Cell. 2004; 14:355–362. 15125838 10.1016/s1097-2765(04)00210-2

[26] Marin-Gonzalez A. , Vilhena J.G., Perez R., Moreno-Herrero F. A molecular view of DNA flexibility. Q. Rev. Biophys. 2021; 54:e8. 34225835 10.1017/S0033583521000068

[27] Basu A. , Bobrovnikov D.G., Ha T. DNA mechanics and its biological impact. J. Mol. Biol. 2021; 433:166861. 33539885 10.1016/j.jmb.2021.166861

[28] Basu A. , Bobrovnikov D.G., Qureshi Z., Kayikcioglu T., Ngo T.T.M., Ranjan A., Eustermann S., Cieza B., Morgan M.T., Hejna M. et al. Measuring DNA mechanics on the genome scale. Nature. 2021; 589:462–467. 33328628 10.1038/s41586-020-03052-3 PMC7855230

[29] Li K. , Carroll M., Vafabakhsh R., Wang X.A., Wang J.-P. DNAcycP: a deep learning tool for DNA cyclizability prediction. Nucleic Acids Res. 2022; 50:3142–3154. 35288750 10.1093/nar/gkac162 PMC8989542

[30] Khan S.R. , Sakib S., Rahman M.S., Samee M..A.H. DeepBend: an interpretable model of DNA bendability. iScience. 2023; 26:105945. 36866046 10.1016/j.isci.2023.105945 PMC9971889

[31] Basu A. , Bobrovnikov D.G., Cieza B., Arcon J.P., Qureshi Z., Orozco M., Ha T. Deciphering the mechanical code of the genome and epigenome. Nat. Struct. Mol. Biol. 2022; 29:1178–1187. 36471057 10.1038/s41594-022-00877-6 PMC10142808

[32] Engel S.R. , Dietrich F.S., Fisk D.G., Binkley G., Balakrishnan R., Costanzo M.C., Dwight S.S., Hitz B.C., Karra K., Nash R.S. et al. The reference genome sequence of *Saccharomyces cerevisiae*: then and now. G3 Bethesda Md. 2014; 4:389–398. 24374639 10.1534/g3.113.008995 PMC3962479

[33] Berardini T.Z. , Reiser L., Li D., Mezheritsky Y., Muller R., Strait E., Huala E. The Arabidopsis information resource: making and mining the “gold standard” annotated reference plant genome. Genesis. 2015; 53:474–485. 26201819 10.1002/dvg.22877 PMC4545719

[34] Goodstein D.M. , Shu S., Howson R., Neupane R., Hayes R.D., Fazo J., Mitros T., Dirks W., Hellsten U., Putnam N. et al. Phytozome: a comparative platform for green plant genomics. Nucleic Acids Res. 2012; 40:D1178–D1186. 22110026 10.1093/nar/gkr944 PMC3245001

[35] Zhang T. , Zhang W., Jiang J. Genome-wide nucleosome occupancy and positioning and their impact on gene expression and evolution in plants. Plant Physiol. 2015; 168:1406–1416. 26143253 10.1104/pp.15.00125 PMC4528733

[36] Zhang T. , Marand A.P., Jiang J. PlantDHS: a database for DNase I hypersensitive sites in plants. Nucleic Acids Res. 2016; 44:D1148–D1153. 26400163 10.1093/nar/gkv962 PMC4702941

[37] Kawakatsu T. , Huang S.C., Jupe F., Sasaki E., Schmitz R.J., Urich M.A., Castanon R., Nery J.R., Barragan C., He Y. et al. Epigenomic diversity in a global collection of *Arabidopsis thaliana* accessions. Cell. 2016; 166:492–505. 27419873 10.1016/j.cell.2016.06.044 PMC5172462

[38] Alonso-Blanco C. , Andrade J., Becker C., Bemm F., Bergelson J., Borgwardt K.M., Cao J., Chae E., Dezwaan T.M., Ding W. et al. 1,135 Genomes reveal the global pattern of polymorphism in *Arabidopsis thaliana*. Cell. 2016; 166:481–491. 27293186 10.1016/j.cell.2016.05.063 PMC4949382

[39] Monroe J.G. , Srikant T., Carbonell-Bejerano P., Becker C., Lensink M., Exposito-Alonso M., Klein M., Hildebrandt J., Neumann M., Kliebenstein D. et al. Mutation bias reflects natural selection in Arabidopsis thaliana. Nature. 2022; 602:101–105. 35022609 10.1038/s41586-021-04269-6 PMC8810380

[40] Castro-Mondragon J.A. , Riudavets-Puig R., Rauluseviciute I., Berhanu Lemma R., Turchi L., Blanc-Mathieu R., Lucas J., Boddie P., Khan A., Manosalva Pérez N. et al. JASPAR 2022: the 9th release of the open-access database of transcription factor binding profiles. Nucleic Acids Res. 2021; 50:D165–D173. 10.1093/nar/gkab1113 PMC8728201 34850907

[41] O’Malley R.C. , Huang S.C., Song L., Lewsey M.G., Bartlett A., Nery J.R., Galli M., Gallavotti A., Ecker J.R. Cistrome and epicistrome features shape the regulatory DNA landscape. Cell. 2016; 165:1280–1292. 27203113 10.1016/j.cell.2016.04.038 PMC4907330

[42] Choi K. , Zhao X., Tock A.J., Lambing C., Underwood C.J., Hardcastle T.J., Serra H., Kim J., Cho H.S., Kim J. et al. Nucleosomes and DNA methylation shape meiotic DSB frequency in *Arabidopsis thaliana* transposons and gene regulatory regions. Genome Res. 2018; 28:532–546. 29530928 10.1101/gr.225599.117 PMC5880243

[43] Naish M. , Alonge M., Wlodzimierz P., Tock A.J., Abramson B.W., Schmücker A., Mandáková T., Jamge B., Lambing C., Kuo P. et al. The genetic and epigenetic landscape of the Arabidopsis centromeres. Science. 2021; 374:eabi7489. 34762468 10.1126/science.abi7489 PMC10164409

[44] Shrikumar A. , Greenside P., Kundaje A. Learning important features through propagating activation differences. Proceedings of the 34th International Conference on Machine Learning. 2017; PMLR 3145–3153.

[45] Shrikumar A. , Tian K., Avsec Ž., Shcherbina A., Banerjee A., Sharmin M., Nair S., Kundaje A. Technical note on transcription factor motif discovery from importance scores (TF-MoDISco) version 0.5.6.5. 2020; arXiv doi:30 April 2020, preprint: not peer reviewed https://arxiv.org/abs/1811.00416.

[46] Zaborowski A.B. , Walther D Determinants of correlated expression of transcription factors and their target genes. Nucleic Acids Res. 2020; 48:11347–11369. 33104784 10.1093/nar/gkaa927 PMC7672440

[47] Bailey T.L. , Johnson J., Grant C.E., Noble W.S. The MEME suite. Nucleic Acids Res. 2015; 43:W39–W49. 25953851 10.1093/nar/gkv416 PMC4489269

[48] Grant C.E. , Bailey T.L., Noble W.S. FIMO: scanning for occurrences of a given motif. Bioinformatics. 2011; 27:1017–1018. 21330290 10.1093/bioinformatics/btr064 PMC3065696

[49] Bai L. , Ondracka A., Cross F.R. Multiple sequence-specific factors generate the nucleosome-depleted region on CLN2 promoter. Mol. Cell. 2011; 42:465–476. 21596311 10.1016/j.molcel.2011.03.028 PMC3119483

[50] Lee W. , Tillo D., Bray N., Morse R.H., Davis R.W., Hughes T.R., Nislow C. A high-resolution atlas of nucleosome occupancy in yeast. Nat. Genet. 2007; 39:1235–1244. 17873876 10.1038/ng2117

[51] Peters J.P. , Yelgaonkar S.P., Srivatsan S.G., Tor Y., Maher J. Mechanical properties of DNA-like polymers. Nucleic Acids Res. 2013; 41:10593–10604. 24013560 10.1093/nar/gkt808 PMC3905893

[52] Chua E.Y.D. , Vasudevan D., Davey G.E., Wu B., Davey C.A. The mechanics behind DNA sequence-dependent properties of the nucleosome. Nucleic Acids Res. 2012; 40:6338–6352. 22453276 10.1093/nar/gks261 PMC3401446

[53] Mitchell J.S. , Glowacki J., Grandchamp A.E., Manning R.S., Maddocks J.H. Sequence-dependent persistence lengths of DNA. J. Chem. Theory Comput. 2017; 13:1539–1555. 28029797 10.1021/acs.jctc.6b00904

[54] Kearse M.G. , Wilusz J.E. Non-AUG translation: a new start for protein synthesis in eukaryotes. Genes Dev. 2017; 31:1717–1731. 28982758 10.1101/gad.305250.117 PMC5666671

[55] Molina C. , Grotewold E. Genome wide analysis of Arabidopsis core promoters. Bmc Genomics [Electronic Resource]. 2005; 6:25. 15733318 10.1186/1471-2164-6-25 PMC554773

[56] Haberle V. , Stark A. Eukaryotic core promoters and the functional basis of transcription initiation. Nat. Rev. Mol. Cell Biol. 2018; 19:621–637. 29946135 10.1038/s41580-018-0028-8 PMC6205604

[57] Faiger H. , Ivanchenko M., Haran T.E. Nearest-neighbor non-additivity versus long-range non-additivity in TATA-box structure and its implications for TBP-binding mechanism. Nucleic Acids Res. 2007; 35:4409–4419. 17576671 10.1093/nar/gkm451 PMC1935006

[58] Yella V.R. , Bansal M. DNA structural features of eukaryotic TATA-containing and TATA-less promoters. FEBS Open Bio. 2017; 7:324–334. 10.1002/2211-5463.12166 PMC5337902 28286728

[59] Miele V. , Vaillant C., Aubenton-Carafa Y., Thermes C., Grange T. DNA physical properties determine nucleosome occupancy from yeast to fly. Nucleic Acids Res. 2008; 36:3746–3756. 18487627 10.1093/nar/gkn262 PMC2441789

[60] Craig R.J. , Hasan A.R., Ness R.W., Keightley P.D. Comparative genomics of *Chlamydomonas*. Plant Cell. 2021; 33:1016–1041. 33793842 10.1093/plcell/koab026 PMC8226300

[61] Derelle E. , Ferraz C., Rombauts S., Rouzé P., Worden A.Z., Robbens S., Partensky F., Degroeve S., Echeynié S., Cooke R. et al. Genome analysis of the smallest free-living eukaryote *Ostreococcus**tauri* unveils many unique features. Proc. Natl. Acad. Sci. 2006; 103:11647–11652. 16868079 10.1073/pnas.0604795103 PMC1544224

[62] Jolma A. , Yan J., Whitington T., Toivonen J., Nitta K.R., Rastas P., Morgunova E., Enge M., Taipale M., Wei G. et al. DNA-Binding Specificities of Human Transcription Factors. Cell. 2013; 152:327–339. 23332764 10.1016/j.cell.2012.12.009

[63] Ngo T.T.M. , Yoo J., Dai Q., Zhang Q., He C., Aksimentiev A., Ha T. Effects of cytosine modifications on DNA flexibility and nucleosome mechanical stability. Nat. Commun. 2016; 7:10813. 26905257 10.1038/ncomms10813 PMC4770088

[64] Keeney S. Egel R. , Lankenau D,-H. Spo11 and the Formation of DNA Double-Strand Breaks in Meiosis. Recombination and Meiosis. 2008; 2:Berlin, Heidelberg Springer Berlin Heidelberg 81–123.Genome Dynamics and Stability. 10.1007/7050_2007_026 PMC3172816 21927624

[65] Hwang P.Y.-H. , Hunter N. Mapping meiotic breaks: spo11 oligonucleotides precisely mark the spots. Genome Biol. 2011; 12:111. 21527047 10.1186/gb-2011-12-4-111 PMC3218852

[66] Lis M. , Walther D The orientation of transcription factor binding site motifs in gene promoter regions: does it matter?. Bmc Genomics [Electronic Resource]. 2016; 17:185. 26939991 10.1186/s12864-016-2549-x PMC4778318

[67] Mack D.R. , Chiu T.K., Dickerson R.E. Intrinsic bending and deformability at the T-A step of CCTTTAAAGG: a comparative analysis of T-A and A-T steps within A-tracts11Edited by I. Tinoco. J. Mol. Biol. 2001; 312:1037–1049. 11580248 10.1006/jmbi.2001.4994

[68] Alvey H.S. , Gottardo F.L., Nikolova E.N., Al-Hashimi H.M. Widespread transient Hoogsteen base pairs in canonical duplex DNA with variable energetics. Nat. Commun. 2014; 5:4786. 25185517 10.1038/ncomms5786 PMC4537320

[69] Manning G.S. The persistence length of DNA is reached from the persistence length of its null isomer through an internal electrostatic stretching force. Biophys. J. 2006; 91:3607–3616. 16935960 10.1529/biophysj.106.089029 PMC1630458

[70] Yan J. , Marko J.F. Localized single-stranded bubble mechanism for cyclization of short double helix DNA. Phys. Rev. Lett. 2004; 93:108108. 15447460 10.1103/PhysRevLett.93.108108

[71] Wiggins P.A. , Phillips R., Nelson P.C. Exact theory of kinkable elastic polymers. Phys. Rev. E Stat. Nonlin. Soft Matter Phys. 2005; 71:021909. 15783354 10.1103/PhysRevE.71.021909 PMC3496790

[72] Jeong J. , Kim H.D. Base-pair mismatch can destabilize small DNA loops through cooperative kinking. Phys. Rev. Lett. 2019; 122:218101. 31283336 10.1103/PhysRevLett.122.218101 PMC7819736

[73] Wan J. , Lin J., Zack D.J., Qian J. Relating periodicity of nucleosome organization and gene regulation. Bioinformatics. 2009; 25:1782–1788. 19447785 10.1093/bioinformatics/btp323 PMC2705233

[74] Hu E.-Z. , Lan X.-R., Liu Z.-L., Gao J., Niu D.-K. A positive correlation between GC content and growth temperature in prokaryotes. Bmc Genomics [Electronic Resource]. 2022; 23:110. 35139824 10.1186/s12864-022-08353-7 PMC8827189

[75] Geggier S. , Vologodskii A. Sequence dependence of DNA bending rigidity. Proc. Natl. Acad. Sci. U.S.A. 2010; 107:15421–15426. 20702767 10.1073/pnas.1004809107 PMC2932579

[76] El Hassan M.A. , Calladine C.R. Conformational characteristics of DNA: empirical classifications and a hypothesis for the conformational behaviour of dinucleotide steps. Philos. Trans. Math. Phys. Eng. Sci. 1997; 355:43–100.

[77] Nalabothula N. , Xi L., Bhattacharyya S., Widom J., Wang J.-P., Reeve J.N., Santangelo T.J., Fondufe-Mittendorf Y.N. Archaeal nucleosome positioning in vivo and in vitro is directed by primary sequence motifs. Bmc Genomics [Electronic Resource]. 2013; 14:391. 23758892 10.1186/1471-2164-14-391 PMC3691661

[78] Laursen S.P. , Bowerman S., Luger K. Archaea: the final frontier of chromatin. J. Mol. Biol. 2021; 433:166791. 33383035 10.1016/j.jmb.2020.166791 PMC7987875

[79] Yang L. , Orenstein Y., Jolma A., Yin Y., Taipale J., Shamir R., Rohs R. Transcription factor family-specific DNA shape readout revealed by quantitative specificity models. Mol. Syst. Biol. 2017; 13:910. 28167566 10.15252/msb.20167238 PMC5327724

[80] Drost J.B. , Lee W.R. Biological basis of germline mutation: comparisons of spontaneous germline mutation rates among drosophila, mouse, and human. Environ. Mol. Mutagen. 1995; 25:48–64. 7789362 10.1002/em.2850250609

[81] Bennett E.P. , Petersen B.L., Johansen I.E., Niu Y., Yang Z., Chamberlain C.A., Met Ö., Wandall H.H., Frödin M. INDEL detection, the ‘Achilles heel’ of precise genome editing: a survey of methods for accurate profiling of gene editing induced indels. Nucleic Acids Res. 2020; 48:11958–11981. 33170255 10.1093/nar/gkaa975 PMC7708060

[82] Gorbunova V. , Levy A.A. How plants make ends meet: DNA double-strand break repair. Trends Plant Sci. 1999; 4:263–269. 10407442 10.1016/s1360-1385(99)01430-2

[83] Lippert M.J. , Kim N., Cho J.-E., Larson R.P., Schoenly N.E., O’Shea S.H., Jinks-Robertson S Role for topoisomerase 1 in transcription-associated mutagenesis in yeast. Proc. Natl. Acad. Sci. U.S.A. 2011; 108:698–703. 21177427 10.1073/pnas.1012363108 PMC3021083

[84] Reijns M.A.M. , Parry D.A., Williams T.C., Nadeu F., Hindshaw R.L., Rios Szwed D.O., Nicholson M.D., Carroll P., Boyle S., Royo R. et al. Signatures of TOP1 transcription-associated mutagenesis in cancer and germline. Nature. 2022; 602:623–631. 35140396 10.1038/s41586-022-04403-y PMC8866115

[85] Jiang W.-J. , Hu C., Lai F., Pang W., Yi X., Xu Q., Wang H., Zhou J., Zhu H., Zhong C. et al. Assessing base-resolution DNA mechanics on the genome scale. Nucleic Acids Res. 2023; 51:9552–9566. 37697433 10.1093/nar/gkad720 PMC10570052

