# Supplementary material for: Predictions of DNA mechanical properties at a genomic scale reveal potentially new functional roles of DNA flexibility
Source: NAR Genom Bioinform. 2023 Nov 6;5(4):lqad097. doi: 10.1093/nargab/lqad097 (PMC10632188; doi:10.1093/nargab/lqad097)
Supplement: lqad097_Supplemental_File [file lqad097_supplemental_file.pdf]

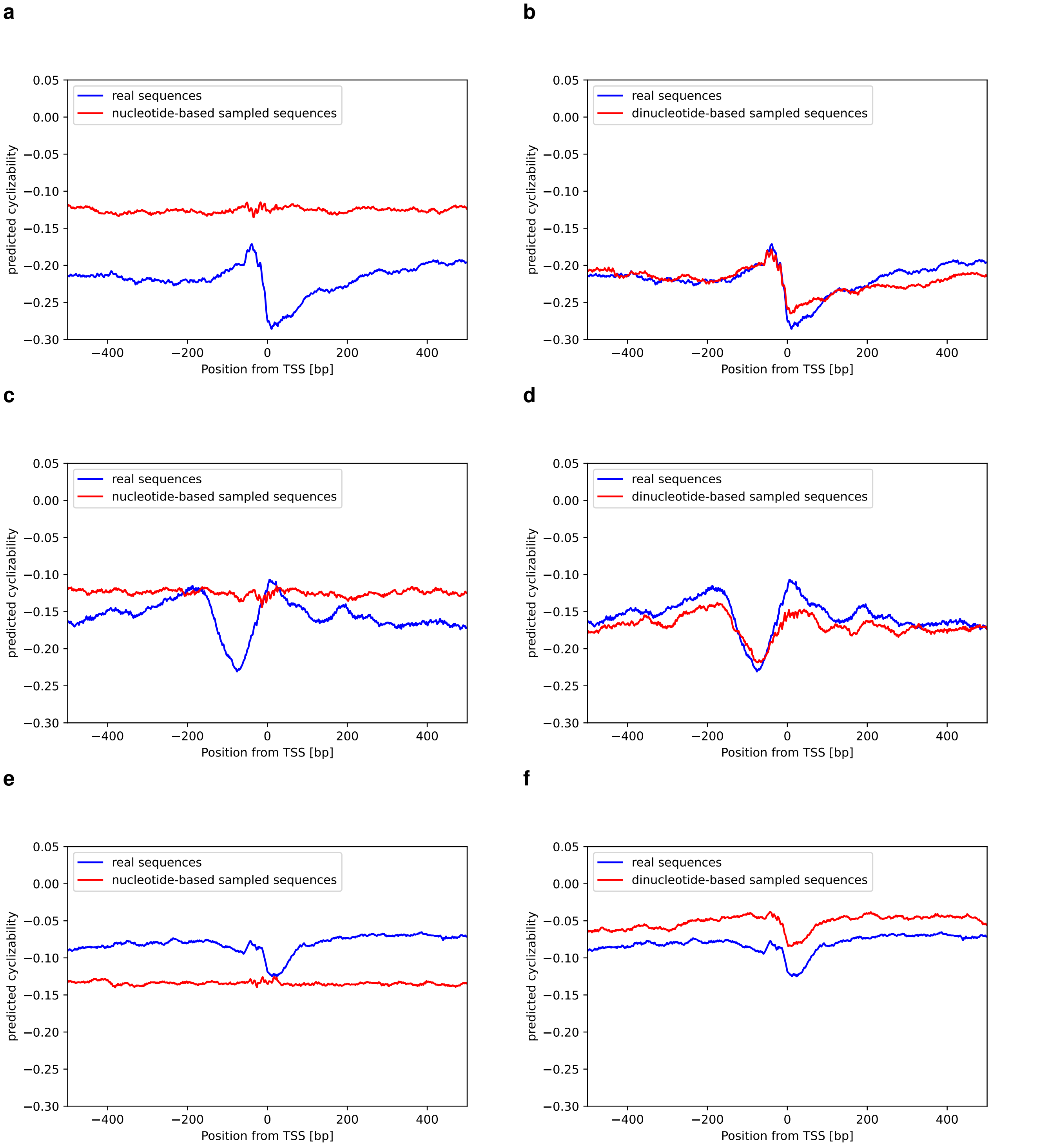

Supplementary Figure S1. Effects of nucleotide and dinucleotide composition on cyclizability.
Mean predicted cyclizability around the TSS of all annotated genes containing a 5' UTR of compared to sampled sequences from the average base composition at each position and sampled sequences from the average dinucleotide composition at each position in **(a)** and **(b)** *Arabidopsis thaliana*, **(c)** and **(d)** *Saccharomyces cerevisiae*, **(e)** and **(f)** *Homo sapiens*, respectively.

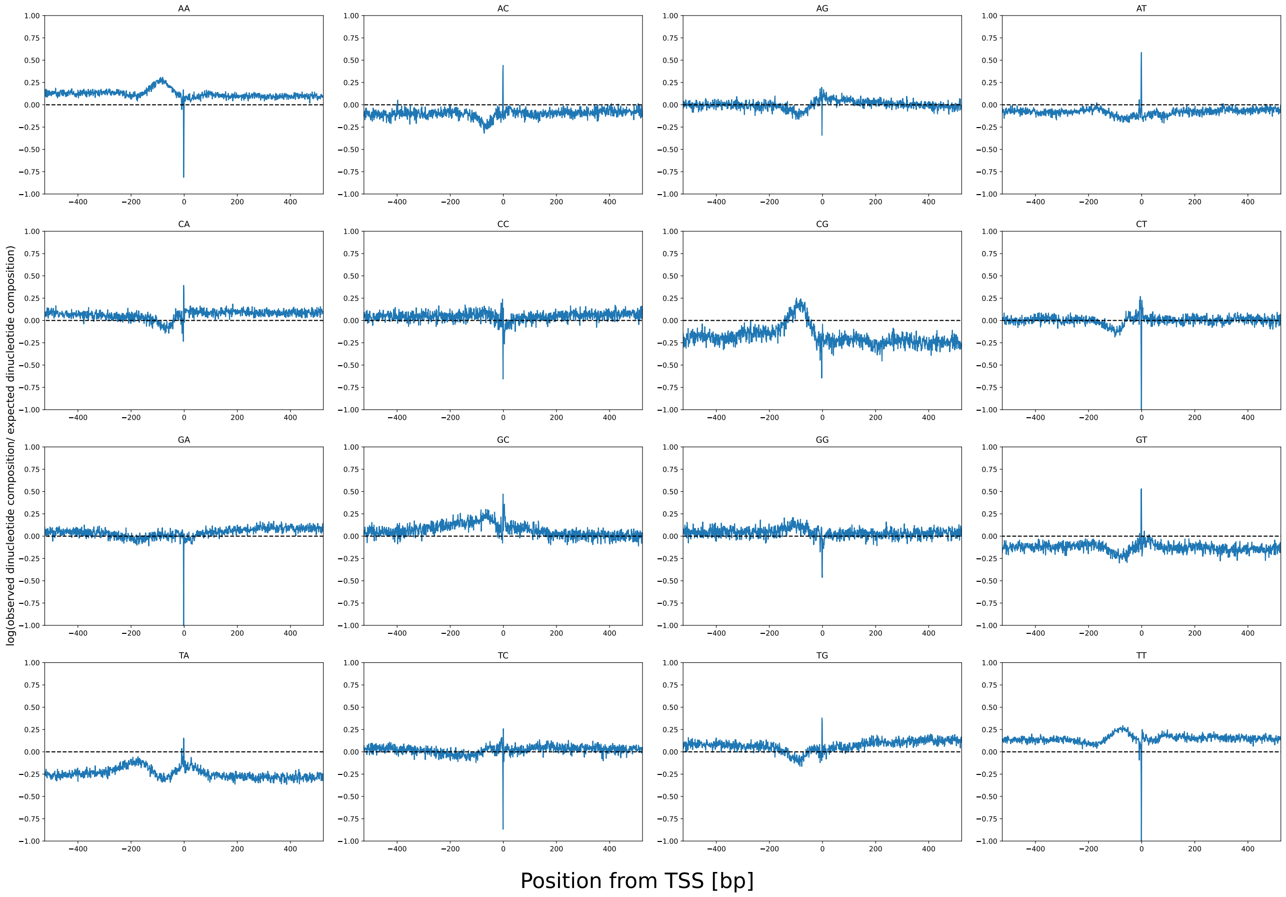

Supplementary Figure S2. Relative dinucleotide composition around the TSS in *S. cerevesia*.
Natural logarithm of the ratio between the observed dinucleotide composition and the expected dinucleotide composition based on nucleotide frequency at each position in *Saccharomyces cerevisiae*.

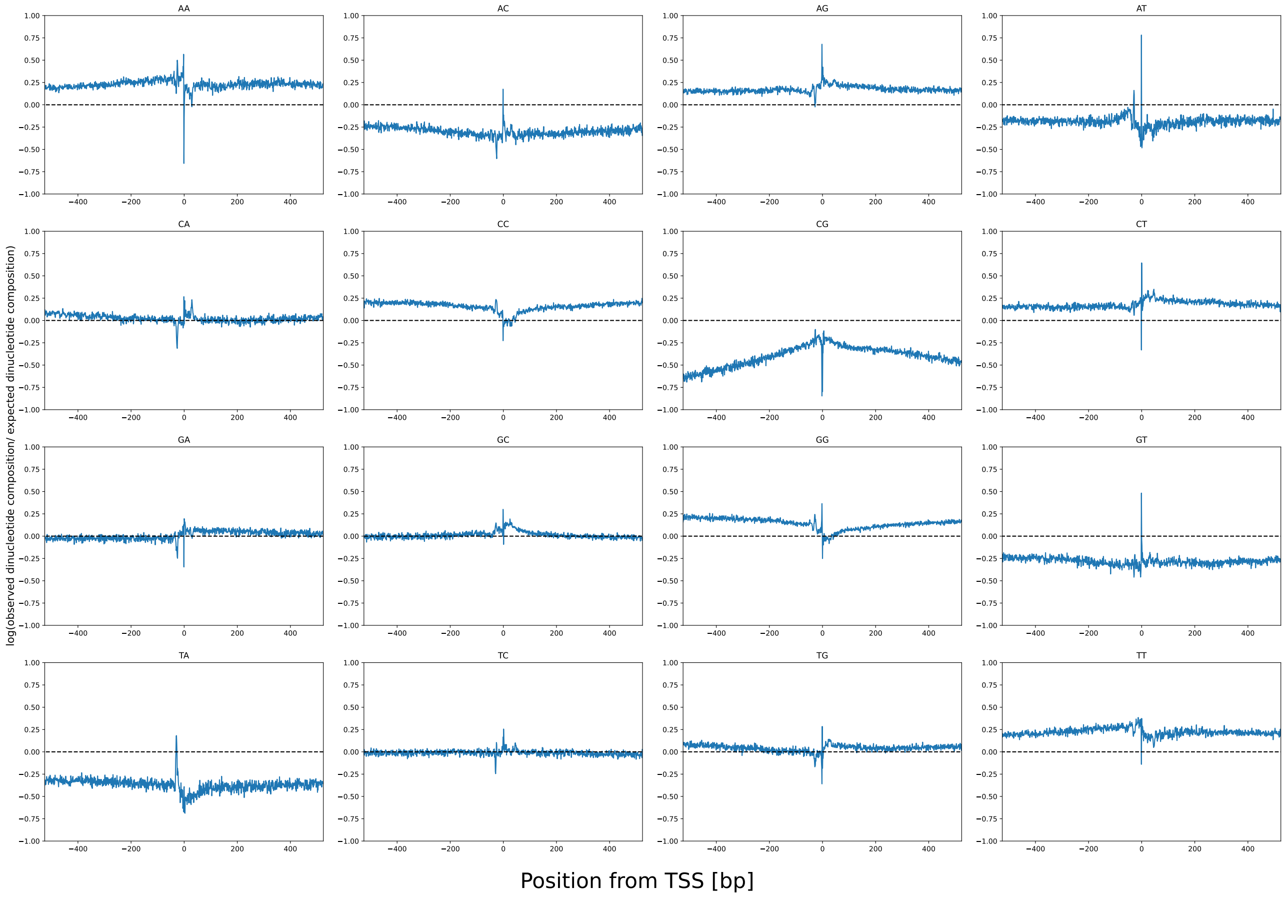

Supplementary Figure S3. Relative dinucleotide composition around the TSS in *Homo sapiens*. Natural logarithm of the ratio between the observed dinucleotide composition and the expected dinucleotide composition based on nucleotide frequency at each position in *Homo sapiens*.

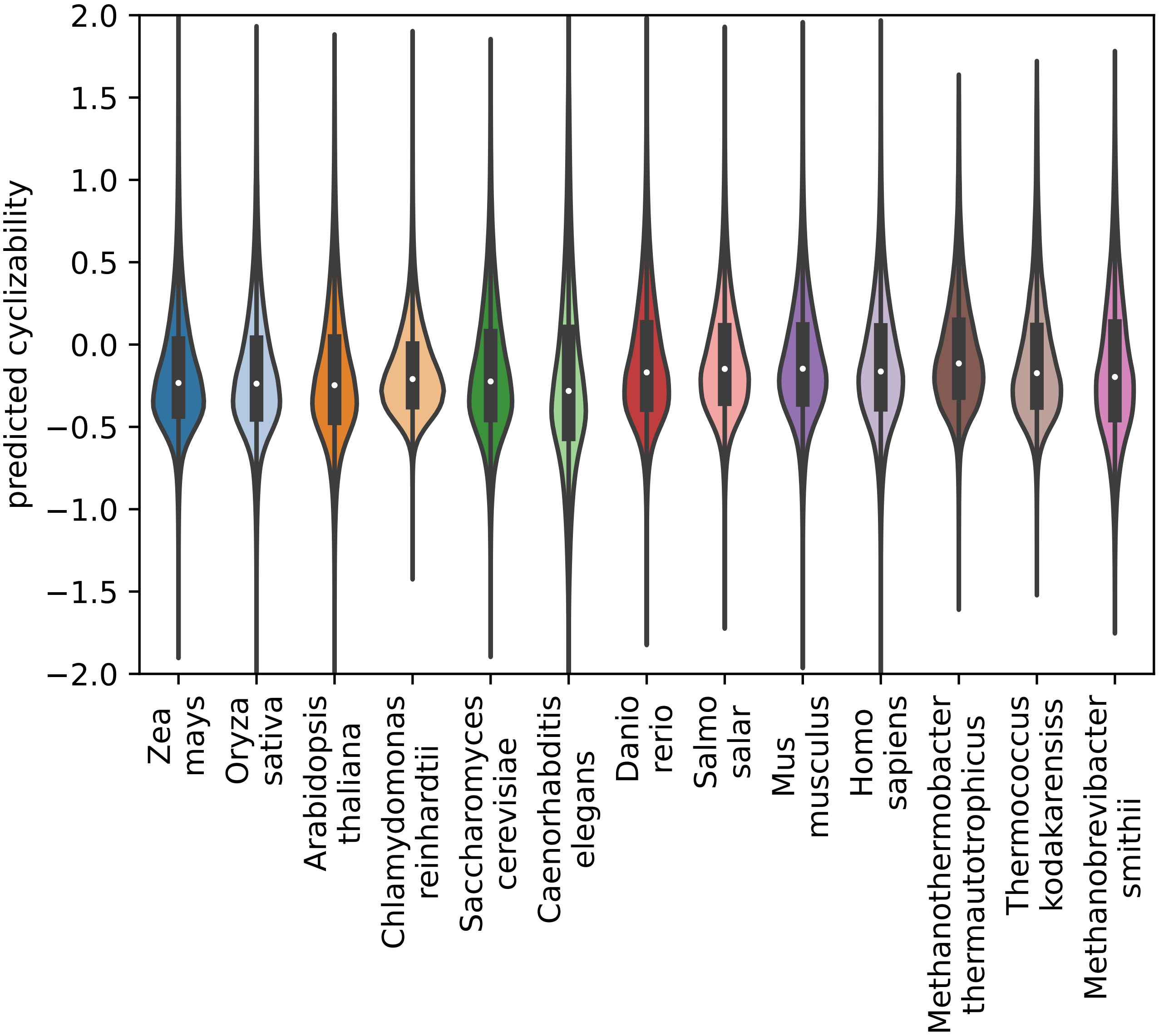

Supplementary Figure S4. Distribution of predicted cyclizability among different species.
